# Thermodynamic Stability and Hydrogen Bonds in Mixed Halide Perovskites

**DOI:** 10.1021/acs.jpclett.6c00523

**Published:** 2026-04-09

**Authors:** Liz Camayo-Gutierrez, Javiera Ubeda, Ana L. Montero-Alejo, Ricardo Grau-Crespo, Eduardo Menéndez-Proupin

**Affiliations:** † Departamento de Física, Facultad de Ciencias Naturales, Matemática y del Medio Ambiente (FCNMM), Universidad Tecnológica Metropolitana, José Pedro Alessandri 1242, Ñuñoa, 7800002 Santiago, Chile; ‡ School of Engineering and Materials Science, Queen Mary University of London, Mile End Road, London E1 4NS, U.K.; ¶ Departamento de Física Aplicada I, Escuela Politécnica Superior, Universidad de Sevilla, Seville E-41011, Spain

## Abstract

The stability of mixed halide perovskites against phase separation is crucial for their optoelectronic applications yet difficult to rationalize due to the interplay of enthalpic, configurational, and dynamical effects. Here we present a simple thermodynamic framework for multicomponent halide perovskites of composition (FAPbI_3_)_1-*x*_(APbY_3_)_*x*_ with A= MA or Cs and Y = I or Br, based on *ab initio* molecular dynamics for *x* = 0, 1/8, and 1. By decomposing the free energy of mixing into enthalpic, configurational, and rotational entropic contributions, we show that although the enthalpy of mixing is small, ranging from nearly zero to moderately positive (+1.06 kJ mol^–1^ at *x*=1/8), the solid solutions are thermodynamically stable against phase separation due to the large configurational entropy (*T*Δ*S*_mix_^conf^ ≈ 1.10–4.39 kJ mol^–1^ at 350 K) associated with random substitution on cation and halide sublattices. Mixing reduces the rotational entropy of the organic cations, partially offsetting the configurational stabilization (*T*Δ*S*_mix_^rot^ ≈ −0.35 to –0.14 kJ mol^–1^). However, within our model, this rotational penalty is not sufficient to overcome the configurational driving force, and a curvature analysis within a regular-solution model does not predict a miscibility gap for any of the mixing channels considered. Analysis of hydrogen-bond dynamics shows that MA–Y interactions are more persistent than FA–Y interactions, while the dominant FA-donated N–H···I hydrogen bonds remain nearly composition-invariant. Cs-containing mixtures, in which Cs^+^ forms no hydrogen bonds, can nevertheless be thermodynamically stable. These results demonstrate that hydrogen bonding does not control thermodynamic stability in mixed halide perovskites. Instead, phase stability is governed by the balance between strong configurational entropy and a smaller, systematically destabilizing rotational-entropy correction.

## Graphical abstract

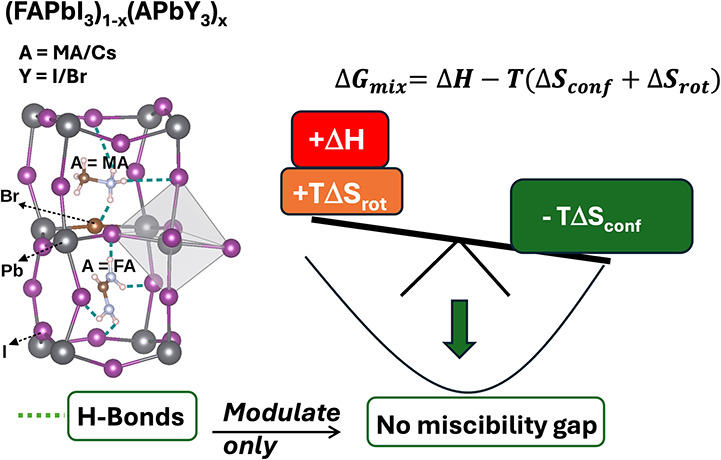

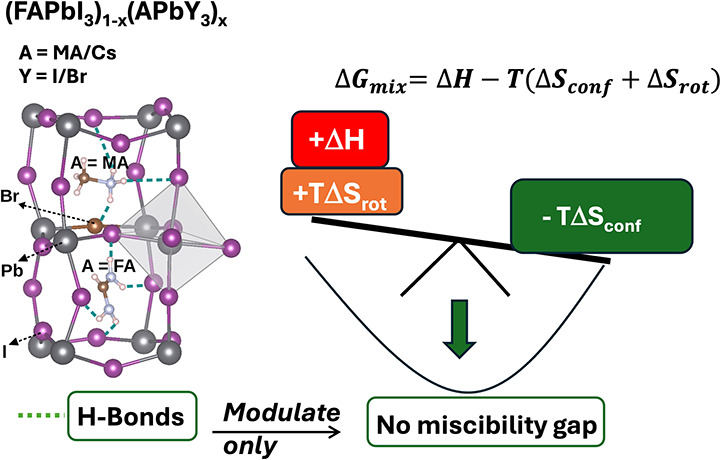

Hybrid organic–inorganic halide perovskites (HOIHP) have attracted intense interest due to their wide range of optoelectronic applications, including in photovoltaics,^1,2^ light-emission devices,^3^ photodetectors,^4,5^ field-effect transistors,^6^ and photocatalysts.^7,8^ Perovskite solar cells (PSCs), employing HOIHP as light absorbers, have reached certified power conversion efficiencies (PCEs) of 27%,^9^ and are promising candidates for tandem silicon-perovskite architectures.

The perovskite structure, derived from CaTiO_3_, is described by the general formula ABY_3_, where the B-site is surrounded by a three-dimensional network of corner-sharing Y_6_ octahedra, and the A-site cation occupies the cavities created by this framework (Figure 1). In HOIHP, the size of the A-site cation is constrained by the Goldschmidt tolerance factor (0.8–1.0),^10^ which limits the most commonly used organic cations to methylammonium (MA = CH_3_NH_3_^+^) and formamidinium (FA = HC­(NH_2_)_2_^+^), while larger cations lead to lower-dimensional structures.^11–14^

**Figure 1:**
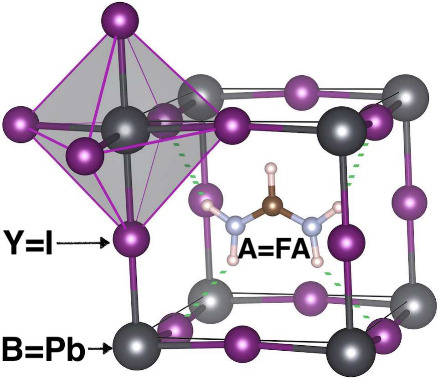
Unit cell of cubic perovskite FAPbI_3_. Iodide anions outside unit cell are added at top left corner to show a PbI_6_ octahedron. Green dotted lines indicate hydrogen bonds.

Early PSCs were based on MAPbI_3_ and FAPbI_3_;^15,16^ however, MAPbI_3_ is prone to decomposition and FAPbI_3_ undergoes detrimental phase transitions at room temperature. Significant improvements in stability and performance have therefore been achieved through alloying.^17–20^ In mixed-cation HOIHP, the A-site is occupied by combinations of FA, MA, and/or Cs, while the B- and Y-sites may also be substituted to reduce toxicity (e.g., Pb/Sn) or tune the band gap and stability (I/Br/Cl). In particular, A-site alloying provides a means to fine-tune the lattice parameters and mitigate interfacial strain in devices.

Hydrogen bonds (HBs) can form in HOIHP between the hydrogens of the organic cations and the surrounding halide anions (Figure 1). According to IUPAC definition,^21^ “the hydrogen bond is an attractive interaction between a hydrogen atom from a molecule or a molecular fragment X–H in which X is more electronegative than H, and an atom or a group of atoms in the same or a different molecule, in which there is evidence of bond formation". In HOIHP, two main types of HBs can occur:^22,23^ N–H···Y and C–H···Y, where Y = Br, Cl, or I. These interactions have been proposed to influence structural stability,^24^ ion migration,^25^ and vibrational properties.

However, HBs in HOIHP are unstable, breaking and reforming on picosecond time scales^26^ even at temperatures as low as 50 K,^27^ where the organic cations do not rotate. The associated activation energies in MAPbBr_3_ are small and comparable to thermal fluctuations,^27,28^ suggesting that individual HBs are weak and transient. While these interactions provide important insight into the local coupling between organic and inorganic sublattices, their actual role in determining the thermodynamic stability of mixed HOIHP remains unclear.

Previous computational studies of FA–Cs systems have shown that Cs incorporation can induce coherent octahedral tilting, slightly restrict FA rotations, and modify noncovalent interactions within the lattice.^29,30^ These effects have been associated with enhanced structural stability. However, most available studies on mixed A-site halide perovskites primarily focus on structural, electronic, or phase-stability aspects.^31–33^ More explicit thermodynamic analyses have recently been reported for related hybrid-perovskite problems, including molecular-dynamics-based studies of entropy changes associated with orientational disorder and phase transitions, as well as computational investigations of free energies of mixing in halide-alloyed perovskites.^34–36^ Related first-principles studies have also addressed thermodynamic stability in other perovskite and perovskite-inspired materials.^37–40^ Nevertheless, an explicit finite-temperature decomposition of the mixing thermodynamics of hybrid perovskite solid solutions into enthalpic, configurational, and rotational contributions has not been reported before.

Although HBs are often discussed as stabilizing interactions in hybrid perovskites, their actual role in the thermodynamic stability of mixed solid solutions remains unclear because free-energy stabilization may also arise from configurational and rotational entropy. Separating HB effects from these entropic contributions is therefore necessary to determine whether HBs are a primary driving force for mixing or a secondary response to the disordered local environment. This distinction provides a more mechanistic understanding of mixed perovskite stability than considering HB dynamics alone.

In this work, we address this open question by performing a combined thermodynamic, structural, and dynamical study of A-site and A+Y-site mixed halide perovskites. Using ab initio molecular dynamics (AIMD), we compute the enthalpy (Δ*H*_mix_), configurational and rotational entropy ( $\Delta S_{\mathrm{mix}}^{\mathrm{conf}}$ , $\Delta S_{\mathrm{mix}}^{\mathrm{rot}}$ ), and effective Gibbs free energy $\left(\Delta G_{\mathrm{mix}}^{\mathrm{tot}}\right)$ of mixing at 350 K for a series of pure and mixed compositions, including FA/MA and FA/Cs alloys at a 7/8 FAPbI_3_ ratio with simultaneous halide substitution. In parallel, we analyze the lifetimes and rebonding probabilities of N–H···Y HBs using time correlation functions, focusing on the effects of system composition.

In our simulations, all mixed perovskites were modeled in the cubic perovskite framework. We adopt a 4 × 4 × 4 supercell size, which was used and validated in our previous AIMD study of (FAPbI_3_)_1–*x*_(MAPbBr_3_)_*x*_, where *x* = 1/8, containing 64 A sites, 64 Pb sites, and 192 halide sites.^41^ Atomic-scale disorder in the mixed configurations was generated using a special quasirandom structure (SQS)-based protocol as in ref 41, selecting configurations that reproduce near-random short-range correlations on the mixed sublattices and near-binomial local-environment statistics. Molecular A-site cations (when present) were then explicitly introduced with randomized orientations. Using the same protocol and supercell size, here we generated three additional mixed perovkites at the mixing fraction 1/8, differing by which sublattices are disordered: (i) A mix (cation-only): (FAPbI_3_)_7/8_(MAPbI_3_)_1/8_, (ii) Y mix (halide-only): (FAPbI_3_)_7/8_(FAPbBr_3_)_1/8_, and (iii) A­(Cs)+Y mix (cation-halide): (FAPbI_3_)_7/8_(CsPbBr_3_)_1/8_. In all cases, the Pb sublattice is fully occupied, and the SQS optimization targets only the sublattices that are compositionally mixed (A, Y, or both), ensuring comparable cell size and sampling across systems.

AIMD simulations were carried out with the same DFT/MD setup (thermostatting protocol, time step, basis sets, pseudopotentials, and dispersion treatment) as in ref 41; full computational details are provided in the Supporting Information. In addition to the mixed supercells described above, we also simulated the corresponding pure end members for each mixing family to provide reference baselines. All trajectories were generated under *NVT* conditions at *T* = 350 K, and each system was propagated for 18 ps of production dynamics after thermalization. These equilibrated production trajectories are used as input for the thermodynamic and hydrogen-bond analyses reported below.

The enthalpy of mixing per formula unit was obtained from equilibrium AIMD trajectories as

1 $$\Delta H_{\mathrm{mix}}\left(x\right)=\left\langle U_{\mathrm{mix}}\left(x\right)\right\rangle -\left(1-x\right)\left\langle U_{A}\right\rangle -x\left\langle U_{B}\right\rangle$$

where ⟨*U*⟩ denotes a time average of the internal energy per formula unit over the production portion of each trajectory (same *T* and ensemble for mixed and reference systems). Uncertainties were estimated from the energy time series by accounting for equilibration and time correlation, using an effective number of independent samples *N*_eff_ to compute the standard error of the mean, SEM (U) = $\sigma /\sqrt{N_{\mathrm{eff}}}$ , σ being the standard deviation. The uncertainty in Δ*H*_mix_ was then obtained by standard error propagation from the SEMs of the mixed and reference energies and is reported as one standard deviation.

The configurational entropy of mixing was evaluated in the ideal disorder limit, following the same assumption as in ref 41. For binary mixing on a given sublattice (A or Y), the contribution per formula unit is

2 $$\Delta S_{\mathrm{mix}}^{\mathrm{conf}}\left(x\right)=-n_{s}k_{B}\left[x\ln x+\left(1-x\right)\;\ln\left(1-x\right)\right]$$

where *n*_*s*_ is the number of mixed sites per ABY_3_ formula unit. Thus, *n*_*s*_ = *n*_*A*_ = 1 for A-mixing, *n*_*s*_ = *n*_*Y*_ = 3 for Y-mixing, and *n*_*s*_ = *n*_*A*_ + *n*_*Y*_ = 4 for simultaneous A+Y mixing, consistent with the expression used in ref 41. This expression corresponds to the ideal disorder limit. The SQS structures reproduce the correlation functions of an ideally disordered alloy but do not explicitly account for possible short-range ordering (SRO). If present, SRO would reduce both Δ*H*_mix_ and Δ*S*_mix_; however, in highly disordered solid solutions these corrections are typically small and tend to partially compensate in the free energy of mixing, so the regular solution description remains a reasonable approximation.^42,43^

Because molecular A-site cations (FA and MA) can rotate within the inorganic cage, the total Gibbs free energy of the mixed perovskites includes an orientational entropy contribution $\Delta G_{\mathrm{mix}}^{\mathrm{tot}}=\Delta H_{\mathrm{mix}}-T\left(\Delta S_{\mathrm{mix}}^{\mathrm{conf}}+\Delta S_{\mathrm{mix}}^{\mathrm{rot}}\right)$ per formula unit. When two distinct cations mix, their reorientational dynamics and thus their rotational entropies may change. The rotational entropy of mixing, $\Delta S_{\mathrm{mix}}^{\mathrm{rot}}$ , measures the difference between the orientational entropy of the mixture and the compositionally–weighted entropies of the pure end members. $\Delta S_{\mathrm{mix}}^{\mathrm{rot}}$ can be evaluated as a species–weighted sum of estimated rotational entropy changes.

3 $$\Delta S_{\mathrm{mix}}^{\mathrm{rot}}\left(x\right)=\underset{\alpha }{\sum }x_{\alpha }\Delta S_{rot}^{\alpha },,\Delta S_{rot}^{\alpha }\approx -\gamma k_{B}\ln\left(\frac{\tau_{\alpha }^{mix}}{\tau_{\alpha }^{pure}}\right)$$

where *x*_α_ is the molar fraction of species α on the A sublattice, and τ_α_ is the corresponding rotational correlation time. The nondimensional factor γ = 0.185 is obtained from the classical hindered-rotor model with a cosine potential,^44^ by linearizing the entropy-loss as a function of the rotational barrier. This approach allows us to estimate the rotational contribution to the mixing entropy without the long simulations required for an explicit rotational entropy calculation (from the distribution of orientations of each cation, which is difficult to converge). Equation 3 implies that when there is no change in the rotational correlation times between the pure systems and the mixtures, there is no rotational contribution to the mixing entropy. But if, as it happens here, the cation rotation is more hindered in the solid solution than in the pure compounds, the correlation time will be longer in the former, and the contribution to the mixing entropy will be negative. The full derivation of eq 3 and its assumptions are provided in the Supporting Information. The correlation times were obtained from normalized orientational autocorrelation functions *C*(*t*) of the main A-molecular axes computed from the AIMD trajectories using the TRAVIS code.^45^ The *C*(*t*) definition, together with details of our operational determination of the correlation time as the orientational half-time τ ≡ *t*_1/2_ (defined as the first time such that *C*(*t*_1/2_) = 0.5), are also provided in the Supporting Information.

The statistics of HBs were collected from MD trajectories using TRAVIS as well. In particular, we computed HB populations and time-correlation descriptors, including combined distribution functions (CDF) and aggregate correlation functions (ACF), to characterize HB geometries and lifetimes in the mixed and pure systems. The CDFs, shown in Figures S4 and S5 in Supporting Information, are two-dimensional histograms of the frequency of a pair of parameters. In the CDFs here presented, that pair of parameters comprises the HB H–Y distance and the N–H–Y angle. The ACF gives the probability that a link between two molecules, e.g., MA and Br linked by a HB, persists during time *t*. The continuous ACF can be cast as

4 $$C_{C}^{HB}\left(t\right)=\frac{A}{N_{1}N_{2}}\overset{N_{1}}{\underset{i=1}{\sum }}\overset{N_{2}}{\underset{j=2}{\sum }}{\left\langle \beta_{ij}\left(t^{\prime }\right)\;{\mathrm{\beta ̃}}_{ij}\left(t^{\prime }+t\right)\right\rangle }_{t\prime }$$

where *i* and *j* run across all molecules of a given species, *i* for an organic cation and *j* for the halogen, respectively. The function β_*ij*_(*t*′) = 1 if the link exists at time *t*′ or 0 if not. The function ${\mathrm{\beta ̃}}_{ij}\left(t^{\prime }+t\right)=1$ if the link exists during the whole time interval (*t*′, *t*′ + *t*). ⟨···⟩_*t*′_ represents the average across the time instants *t*′ in the MD simulation. Finally, *A* is a normalization factor to accomplish $C_{C}^{HB}\left(0\right)=1$ . The lifetime of the aggregate is given by the integral^45^

5 $$\tau =2\int_{0}^{\infty }C_{C}^{HB}(t)\;dt$$

The intermittent ACF $C_{I}^{HB}\left(t\right)$ is defined by an expression similar to eq 4 with the function ${\mathrm{\beta ̃}}_{ij}$ replaced by β_*ij*_. Thus, $C_{I}^{HB}\left(t\right)$ gives the probability of the link existence if it was established a time *t* in the past, not requiring the continuous existence at intermediate times.

We now present the calculated thermodynamic trends and hydrogen-bond dynamics.

In previous work on mixed perovskites, we reported that cation reorientation dynamics in (FAPbI_3_)_7/8_(MAPbBr_3_)_1/8_ differ from those of the corresponding pure compounds, although the observed effect was relatively modest.^41^ In the present study, by extending the analysis to additional mixing channels and compositions, we find that changes in molecular reorientation dynamics are significantly more pronounced and strongly dependent on the nature of the mixed sublattices. In particular, A-site mixing and the Cs-containing A+Y mixture exhibit substantial modifications of cation rotation, while more moderate effects persist in Y-site mixing and in (FAPbI_3_)_7/8_(MAPbBr_3_)_1/8_. These observations motivate a quantitative assessment of the role of molecular rotation in the thermodynamics of mixing. To this end, we analyze the reorientation dynamics of representative molecular vectors obtained from molecular dynamics trajectories (Figure 2) and use the resulting rotational correlation times to estimate the rotational entropy change upon mixing.

**Figure 2:**
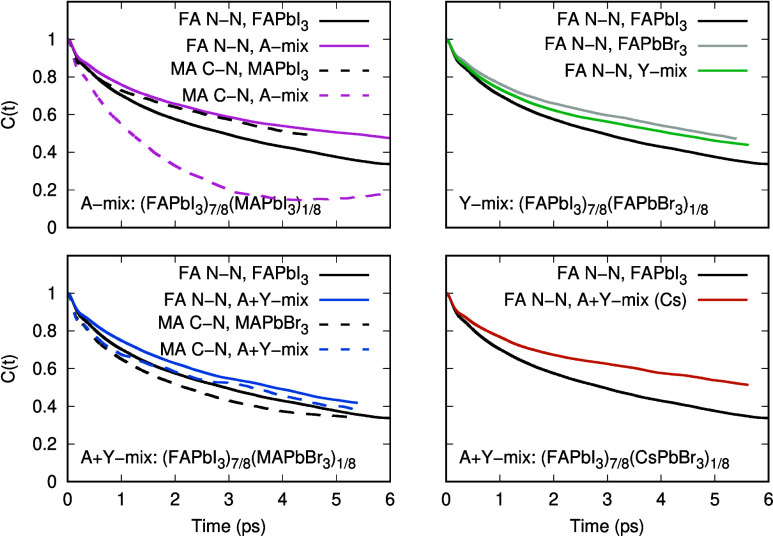
Orientational autocorrelation functions *C*(*t*) for the FA N–N axis and the MA C–N axis in pure and mixed perovskites (A-site, Y-site, and A+Y mixing). Slower decay (larger correlation time) indicates slower reorientation; correlation times are obtained from fits as described in the Supporting Information.

The reorientation dynamics of the FA N–N vector and the MA C–N vector (Figure 2), reveal a composition-dependent modification of molecular rotation. In all mixed compositions, the reorientation of FA is systematically slowed down relative to pure FAPbI_3_, indicating a restriction of its accessible orientational phase space upon mixing. This systematic slowdown indicates that both A-site substitution (FA/MA or FA/Cs) and halide substitution (I/Br) perturb the cage in a way that reduces FA rotational freedom, pointing to a mixing-induced orientational constraint on FA that contributes negatively to $\Delta S_{\mathrm{mix}}^{\mathrm{rot}}$ . In contrast, MA reorientation exhibits a mixing dependence: it is markedly accelerated in (FAPbI_3_)_7/8_(MAPbI_3_)_1/8_, whereas it is slowed in (FAPbI_3_)_7/8_(MAPbBr_3_)_1/8_ relative to MAPbI_3_ and MAPbBr_3_. In line with the above trends, solid-state NMR relaxometry indicates that cation reorientation is strongly affected by the inorganic-lattice symmetry: FA slows near the cubic-tetragonal transition (and may slow down further in mixtures with Cs), while MA shows a ∼2× faster rotation at room temperature in cubic FA_*x*_MA_1–*x*_PbI_3_ compared with MAPbI_3_.^47^

Based on these reorientation dynamics, we estimate the rotational entropy change upon mixing, $\Delta S_{\mathrm{mix}}^{\mathrm{rot}}$ , providing a microscopic basis for the rotational entropy term included in the thermodynamic analysis. The underlying reorientation times τ_α_ (FA N–N and MA C–N), taken in practice as the fit-free half-time *t*_1/2_ from the AIMD autocorrelation functions, are reported in Table S1 of the Supporting Information. Table 1 summarizes the thermodynamic quantities for all mixed perovskites at 350 K, including enthalpies of mixing, the ideal configurational entropy, the rotational entropy change extracted from molecular-dynamics trajectories, and the corresponding total Gibbs free energies $\Delta G_{\mathrm{mix}}^{\mathrm{tot}}$ .

**Table 1:** Thermodynamic Potentials of Mixing (per Formula Unit) for the Studied Perovskites (ABY_3_) at 350 K^a^

Mixing site	Perovskite	Δ*H*_mix_ (kJ mol^–1^)	$\Delta S_{\mathrm{mix}}^{\mathrm{conf}}$ (kJ K^–1^ mol^–1^)	$\Delta S_{\mathrm{mix}}^{\mathrm{rot}}$ (kJ K^–1^ mol^–1^)	$\Delta G_{\mathrm{mix}}^{\mathrm{tot}}$ (kJ mol^–1^)
A	(FAPbI_3_)_7/8_(MAPbI_3_)_1/8_	–0.11(21)	3.13 × 10^–3^	–5.35 × 10^–4^	–1.02(21)
Y	(FAPbI_3_)_7/8_(FAPbBr_3_)_1/8_	+0.70(24)	9.40 × 10^–3^	–4.01 × 10^–4^	–2.45(24)
A+Y	(FAPbI_3_)_7/8_(MAPbBr_3_)_1/8_	+0.91(22)	1.25 × 10^–2^	–4.48 × 10^–4^	–3.32(22)
A+Y	(FAPbI_3_)_7/8_(CsPbBr_3_)_1/8_	+1.06(19)	1.25 × 10^–2^	–9.89 × 10^–4^	–2.98(19)

a The configurational entropy corresponds to the ideal mixing of *N* substitutional sites (A and/or Y), while $\Delta S_{\mathrm{mix}}^{\mathrm{rot}}$ is the loss of rotational entropy estimated from molecular dynamics trajectories (see the Supporting Information for the derivation and assumptions). The total free energy is $\Delta G_{\mathrm{mix}}^{\mathrm{tot}}=\Delta H_{\mathrm{mix}}-T\left(\Delta S_{\mathrm{mix}}^{\mathrm{conf}}+\Delta S_{\mathrm{mix}}^{\mathrm{rot}}\right)$ . Uncertainties correspond to one standard deviation.

A direct comparison of the entropy contributions in Table 1 reveals a clear separation of roles between configurational and rotational entropy. The configurational entropy of mixing is positive and constitutes the dominant stabilizing contribution for all compositions, increasing systematically with the number of mixed sites. In contrast, the rotational entropy is negative for all systems: because $\frac{\tau^{mix}}{\tau^{pure}}>1$ , it acts as a destabilizing contribution that partially offsets the configurational entropy.

To assess the stability of the solid solutions against phase separation, it is not enough to consider the sign of the mixing free energy at a single composition *x*: one must consider the curvature of the mixing free energy as a function of *x*. Extending the AIMD-based thermodynamic analysis to a grid of *x* values is computationally too expensive, but we can analyze the curvature of the mixing Gibbs free energy within the framework of a regular-solution model, parametrized using the computed thermodynamic quantities at *x* = 1/8 (Figure 3). Here *W* is an enthalpic parameter obtained from the bowing of Δ*H*_mix_ = *Wx*(1 – *x*), while λ captures the rotational-entropy correction via $-T\Delta S_{\mathrm{mix}}^{\mathrm{rot}}=T\lambda x\left(1-x\right)$ . With this definition, λ > 0 corresponds to a loss of rotational entropy upon mixing. In this model, a miscibility gap occurs when *W*_eff_ > 2*n*_*s*_ *k*_B_ *T*, where *W*_eff_ = *W* + *Tλ* and *n*_*s*_ is the number of mixed sites per formula unit. This criterion diagnoses the enthalpy/entropy tendency toward phase separation within the regular-solution approximation.

**Figure 3:**
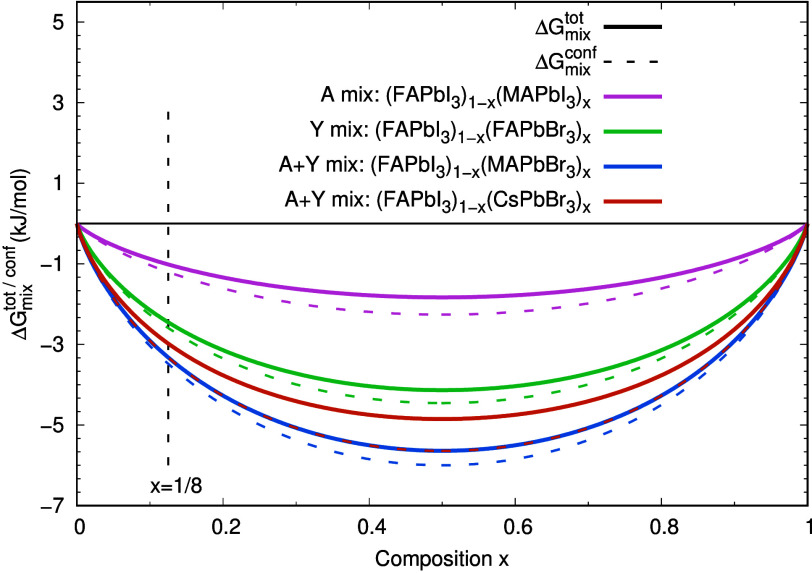
Thermodynamic bowing curves for $\Delta G_{\mathrm{mix}}^{\mathrm{tot}}$ (solid lines) and $\Delta G_{\mathrm{mix}}^{\mathrm{conf}}$ (dashed lines) at 350 K for A-site, Y-site, and A+Y-site mixed perovskites. The vertical dashed line marks the simulated composition *x* = 1/8.

Although $\Delta G_{\mathrm{mix}}^{\mathrm{tot}}$ is negative at the simulated composition for all systems, the propensity for macroscopic phase separation is governed by the curvature of Δ*G*_mix_(*x*). Quantitatively, for all mixing channels considered here the curvature criterion does not predict a miscibility gap at 350 K. Specifically, we obtain *W*_eff_ = 7.67 kJ mol^–1^ for (FAPbI_3_)_7/8_(FAPbBr_3_)_1/8_ (Y-mix), 0.74 kJ mol^–1^ for (FAPbI_3_)_7/8_(MAPbI_3_)_1/8_ (A-mix), 9.72 kJ mol^–1^ for (FAPbI_3_)_7/8_(MAPbBr_3_)_1/8_ (A+Y mix), and 12.88 kJ mol^–1^ for the Cs-containing A+Y mixture (FAPbI_3_)_7/8_(CsPbBr_3_)_1/8_. All values remain below the corresponding thresholds, indicating that within this regular-solution analysis, the free-energy curves remain convex and the solid solutions are thermodynamically stable against macroscopic phase separation.

The rotational entropy contribution estimated in this work is temperature-specific, as it is derived from molecular dynamics simulations performed at 350 K. A quantitative extrapolation to other temperatures would therefore require additional simulations. Nevertheless, some qualitative trends can be inferred. The dominant configurational entropy contribution scales linearly with temperature, so the entropic stabilization of the mixed phases is expected to increase at higher temperatures, provided the perovskite phase remains structurally stable and below the decomposition temperature. At lower temperatures, the entropic contribution becomes weaker and, in principle, a miscibility gap could appear, as predicted by regular-solution descriptions of solid solutions. A simple extrapolation of the present model suggests that the critical temperature for the onset of such a miscibility gap would lie in the approximate range 130–170 K for the mixed perovskites with positive Δ*H*_mix_. However, hybrid halide perovskites typically undergo structural phase transitions to lower-symmetry phases upon cooling at temperatures above this range. Therefore, within the stability range of the high-symmetry phase considered here, the mixed systems are expected to remain thermodynamically stable.

These trends are consistent with previous atomistic and experimental studies suggesting that mixed-cation and mixed-halide perovskites can form stable solid solutions over broad composition ranges, with configurational entropy and strain accommodation playing central roles in the thermodynamic balance.^31,48^ In the specific case of FA/Cs mixtures, Cs incorporation has been reported to stabilize the perovskite phase by reducing local lattice frustration and promoting a more coherent octahedral tilting pattern.^30^ This overall tendency toward solid-solution stability is also consistent with FA/MA mixing, although MA is often associated with increased dynamic disorder and volatility rather than with the suppression of structural instabilities. In fact, temperature-dependent experiments report FA/MA solid solutions across the full composition range and a cubic phase at high temperature for all *x*.^33^

In our simulations, Y-mixing and A+Y mixing are strongly stabilized by configurational entropy, whereas FA/MA A-site mixing shows a smaller but still negative $\Delta G_{\mathrm{mix}}^{\mathrm{tot}}$ at this composition. The Cs-containing A+Y mixture exhibits the largest rotational-entropy penalty, consistent with more constrained FA dynamics, yet remains thermodynamically favorable and well below the miscibility threshold in the curvature analysis. Overall, these results support a picture in which configurational entropy provides the dominant stabilization, while rotational entropy contributes a systematic, typically destabilizing correction that can modulate the curvature of Δ*G*_mix_(*x*).

Notably, the (FAPbI_3_)_7/8_(MAPbBr_3_)_1/8_ (A+Y mixed) composition exhibits the most favorable (most negative) Δ*G*_mix_(*x*) among the systems studied, consistent with the widespread use of mixed-cation/mixed-halide absorbers in high-performance perovskite solar cells.^18–20^ Mixed FA/MA and I/Br formulations form the basis of many state-of-the-art device recipes, where compositional engineering is commonly rationalized in terms of improved phase purity and enhanced thermodynamic stability driven primarily by configurational entropy rather than strongly exothermic interactions.^49–51^ Our results provide an equilibrium thermodynamic counterpart to this empirical success: simultaneous A- and Y-sublattice disorder maximizes the entropic stabilization and yields a strongly favorable mixing free energy. We emphasize, however, that device record efficiencies also depend critically on crystallization pathways, defect chemistry, and interfaces, and mixed-halide systems may still exhibit light-induced segregation under operating conditions.^50^

Having established that all mixing channels considered here are thermodynamically stable at 350 K (with no miscibility gap predicted within our regular-solution analysis), we next examine whether hydrogen bonding provides a microscopic origin for the observed differences among mixing channels. We address this point by analyzing the lifetimes and rebonding probabilities of the hydrogen bonds.

We consider that a N–H···Y HB (Y = Br, I) is established when two geometrical conditions are simultaneously fulfilled:(1) the distance *d*(H–Y) ≤ 3 Å, and (2) the angle ∠(N–H–Y) is between 135° and 180°. This procedure was established^23^ by analyzing the CDF of *d*(H–Y) and ∠(N–H–Y), for the cases of FAPbI_3_, MAPbBr_3_, and (FAPbI_3_)_7/8_(MAPbBr_3_)_1/8_. That analysis was complemented by calculations of the reduced density gradient of the electronic density. Figure S4–5 in Support Information shows the CDFs for the former compounds and the new compounds here studied. It can be seen that the aforementioned conditions enclose a region of high probability for all the studied materials. Moreover, for each particular HB, e.g., (FA)­N–H···Br,^46^ the appearance of the CDF is the same for all compounds. Hence, we keep these geometrical criteria to determine the dynamical properties of the HBs in these materials.

Figure 4 shows the continuous and intermittent ACF for all the studied materials. The functions in the graphs are displayed up the HB lifetime (*t*/τ = 1). The HB lifetimes τ extracted from the continuous ACF are summarized in Table 2. The intermittent ACF cannot decay to zero because the confinement of the FA and MA cations implies that all broken HBs eventually reform. Nevertheless, the difference between the intermittent and continuous ACF is a measure of the fraction of cation-halogen pairs that rebond after HB breaking instead of evolving to form a HB with different pairs. In particular, the difference between both ACFs at the lifetime is reported as probability difference (PD) in Table 2. Overall, HB lifetimes remain in a narrow subps range across pure and mixed systems, whereas PD and the coordination number *N*_*n*_ mainly reflect local halide availability (notably the low Br fraction in the mixed-halide cases). This indicates that hydrogen bonds adapt to the mixed environment, but do not provide a direct thermodynamic driving force for the distinct mixing stabilities discussed above.

**Figure 4:**
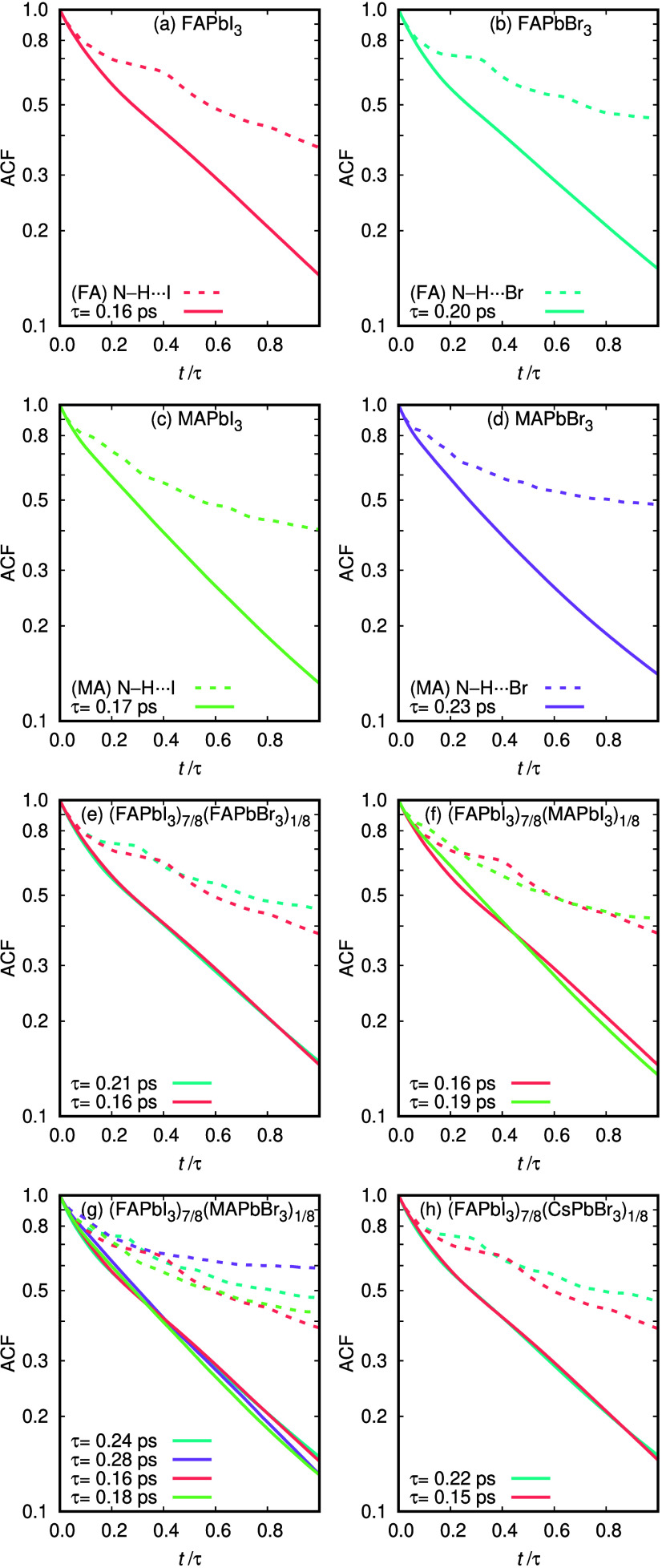
(a)–(h) ACFs of N–H···I and N–H···Br HBs calculated as continuous (continuous line) and intermittent types (dashed line) of the pure and mixed compounds.

**Table 2:** Hydrogen-Bond Lifetimes *τ* between A-Site Cations and Y-Site Anions in Pure and Mixed Perovskites, Obtained from Continuous Time Correlation Functions at 350 K^a^

	(FA) N–H···I^46^			(FA) N–H···Br			(MA) N–H···I			(MA) N–H···Br		
Perovskite	PD^a^	τ (ps)	*N*_*n*_	PD^a^	τ (ps)	*N*_*n*_	PD^a^	τ (ps)	*N*_*n*_	PD^a^	τ (ps)	*N*_*n*_
FAPbI_3_	0.223	0.156	2.0									
FAPbBr_3_				0.298	0.197	2.6						
MAPbI_3_							0.271	0.171	1.7			
MAPbBr_3_										0.312	0.229	2.2
(FAPbI_3_)_7/8_(MAPbI_3_)_1/8_	0.234	0.156	2.0				0.286	0.193	1.8			
(FAPbI_3_)_7/8_(FAPbBr_3_)_1/8_	0.233	0.158	1.7	0.302	0.213	0.4						
(FAPbI_3_)_7/8_(MAPbBr_3_)_1/8_	0.238	0.161	1.7	0.325	0.242	0.4	0.297	0.183	2.0	0.457	0.275	0.2
(FAPbI_3_)_7/8_(CsPbBr_3_)_1/8_	0.233	0.151	1.7	0.312	0.226	0.4						

a PD measures bond reformation probability, and *N*_*n*_ is the average number of bonded neighbors.

b PD = *C*_int_(*t* = τ) – *C*_cont_(*t* = τ), where *C*_int_ and *C*_cont_ are the intermittent and continuous correlation functions, respectively.

The following trends are observed. The (FA)­N–H···I HB has almost the same lifetime (±3–4%) and PD for all compositions. This is probably due to the fact that all compounds have either 100% or 87.5% FA and I content. The number of neighbors (halogen neighbors of FA or FA neighbors of I) is proportional to the iodine content. The number of neighbors is the average number of halogen anions hydrogen-bonded to each FA or MA cation. More significant variations can be observed for the HBs that become minority in mixed compounds. The (FA)­N–H···Br bonds in the pure compound FAPbBr_3_ show longer lifetime and greater number of neighbors than the (FA)­N–H···I in pure FAPbI_3_. In the mixed compounds the number of neighbors decreases strongly because Br content becomes minoritary. The decrease in the number of neighbors correlates with an increase of lifetime and probability difference. In other words, there are fewer bonds, but once established they last longer. This behavior is characteristic of minority–species effects, where reduced local configurational freedom restricts bond exchange and leads to enhanced persistence of the remaining HBs. The same behavior can be appreciated for the (MA)­N–H···Br HBs present in MAPbBr_3_ and (FAPbI_3_)_7/8_(MAPbBr_3_)_1/8_.

Overall, the HBs statistics reveal a consistent hierarchy in both lifetime and rebonding probability: Br-based interactions are more persistent and prone to rebonding than their iodide counterparts, whereas MA–centered bonds display greater orientational memory than those involving FA. Conversely, (FA)­N–H···I HB remain essentially unaffected by the composition, underscoring the intrinsic character of this interaction within the PbI lattice.

The FA/Cs mixture ((FAPbI_3_)_7/8_(CsPbBr_3_)_1/8_) deserves special attention. Its (FA)­N–H···I lifetimes are slightly shorter than in FAPbI_3_, but PD is marginally higher while *N*_*n*_ decreases only modestly. Thus, FA-I interactions in (FAPbI_3_)_7/8_(CsPbBr_3_)_1/8_ break on similar time scales but tend to reform with the same acceptor, indicating a confined and persistent FA-halide environment rather than a weaker HB network. This is consistent with previous ab initio studies reporting restricted cation-lattice dynamics and shorter N–H···I distances upon Cs incorporation.^29,30^ Importantly, these dynamical differences do not systematically track the mixing thermodynamics (Table 1) and do not provide a microscopic explanation for the overall stability of (FAPbI_3_)_7/8_(CsPbBr_3_)_1/8_, reinforcing that hydrogen bonding is not the primary driver of thermodynamic stability.

In the (FAPbI_3_)_7/8_(MAPbBr_3_)_1/8_ solid solution, MA forms more persistent N–H···Y interactions than FA (Table 2), particularly for Br where τ and PD are the largest ones despite the low coordination *N*_*n*_. Thus, the mixed-cation environment supports coexisting HB regimes with distinct persistence and rebonding behavior (FA–I versus MA–I/Br), which can promote local dynamical heterogeneity even though the dominant FA–I lifetime itself remains nearly composition-invariant. This heterogeneity may contribute to local frustration, although it does not map directly onto Δ*H*_mix_ or $\Delta G_{\mathrm{mix}}^{\mathrm{tot}}$ .

Reorientational A-site dynamics (Figure S2, Supporting Information) further support this picture. FA reorientation slows systematically in mixed compositions, whereas MA reorientation is mixing-channel dependent (slower in (FAPbI_3_)_7/8_(MAPbI_3_)_1/8_ but faster in (FAPbI_3_)_7/8_(MAPbBr_3_)_1/8_ relative to the corresponding pure compound). Since (FA)­N–H···I HB lifetimes remain nearly unchanged, the slowdown reflects increased anisotropy and heterogeneity of the local environment rather than stronger individual interactions. The corresponding loss of rotational entropy is small but consistent with the computed $\Delta S_{\mathrm{mix}}^{\mathrm{rot}}$ values. As shown before, these dynamical contributions are secondary to the dominant configurational entropy term that stabilizes mixing.

To test whether HB behavior correlates with broader structural features, we compared HB lifetimes with the Root Mean Squared displacement (RMSD) across compositions (Figure S10 in the Supporting Information). These global descriptors vary only modestly and do not correlate with either HB statistics or mixing enthalpies. A noteworthy exception is the FA/Cs system, where the FA center-of-mass RMSD closely follows that of Cs, indicating a high degree of dynamical compatibility that contrasts with the heterogeneity observed in FA/MA mixtures. These observations reinforce the conclusion that FA/MA and FA/Cs mixtures achieve comparable thermodynamic stability through distinct microscopic pathways, rather than through a dominant HB-based mechanism.

Taken together, these observations indicate that the hydrogen bonding is not the primary thermodynamic driving force for mixing in the perovskite considered here. The dominant FA–I HBs exhibit nearly invariant lifetimes and rebonding probabilities across compositions and therefore do not systematically track either Δ*H*_mix_, $\Delta G_{\mathrm{mix}}^{\mathrm{tot}}$ , or the phase-stability trends inferred from free-energy curvature. Although Br-based and MA-centered interactions are typically longer-lived and can display enhanced rebonding (notably for MA–Br in (FAPbI_3_)_7/8_(MAPbBr_3_)_1/8_), these local dynamical signatures do not translate into systematically more favorable mixing free energies. Conversely, Cs does not form hydrogen bonds, yet the Cs-containing mixture (FAPbI_3_)_7/8_(CsPbBr_3_)_1/8_ remains thermodynamically favorable at *x* = 1/8 and does not exhibit a miscibility gap within our curvature analysis, indicating that hydrogen bonding is not a prerequisite for mixing stability in these systems.

In conclusion, we find that hydrogen bonds are not the primary thermodynamic driving force for the stability of mixed FA/MA/Cs halide perovskites. Although hydrogen bonding is essential to sustain the hybrid framework and modulate local interactions, their lifetimes and rebonding probabilities do not correlate with the calculated Δ*H*_mix_ or Δ*G*_mix_. Instead, stability is dominated by configurational entropy, with lattice strain accommodation, and a systematically destabilizing rotational-entropy correction providing secondary contributions.

Importantly, comparable thermodynamic stability can arise through different microscopic responses. In FA/MA solid solutions, the coexistence of two hydrogen-bonding cations promotes dynamical heterogeneity and local frustration of the HB network. In contrast, FA/Cs systems exhibit a more homogeneous dynamical response due to the absence of HB competition and the greater compatibility of the A-site species. In both cases, the HB network adapts to the mixed environment rather than dictating the thermodynamic outcome.

By combining thermodynamic analysis with HB statistics and cation reorientation dynamics, we show that HBs play a secondary, modulatory role in the stabilization of mixed halide perovskites. These materials instead achieve stability through a subtle interplay of configurational disorder, lattice response, and adaptive local interactions.

Note that our analysis is based on idealized atomistic models and equilibrium thermodynamics. In real materials, additional factors such as defects, strain gradients, illumination-induced segregation, and finite-size effects may influence phase stability. Within these limitations, our calculations indicate that all the solid solutions considered are thermodynamically stable against phase separation, without miscibility gaps at 350 K. Beyond the specific systems studied here, the present thermodynamic framework may also be useful for other mixed hybrid perovskites and related multicomponent molecular solids, where phase stability is controlled not only by mixing enthalpy but also by configurational and orientational contributions. This perspective may help distinguish between local interactions that directly govern thermodynamic stability and those that mainly adapt to the disordered environment.

## Supplementary Material



## Data Availability

The raw AIMD trajectories and the processed data sets derived from them (TRAVIS outputs for hydrogen-bond analysis, cation reorientation dynamics, and mean-square displacement) are available on Zenodo at DOI: 10.5281/zenodo.18604579.

## Notes

(i) Derivation of the rotational-entropy-of-mixing estimator for mixed-cation perovskites (including the operational definition of the reorientation time via the orientational half-time (*t*_1/2_); (ii) computational methodology, (iii) additional cation reorientation dynamics results; (iv) estimation of uncertainties in the calculated thermodynamic functions; (v) regular-solution model analysis of mixing free energies and phase stability; (vi) detailed hydrogen-bond structural and time-correlation analyses; and (vii) root-mean-square displacement (RMSD) analysis (PDF)
