# Supplementary material for: Thermodynamic Stability and Hydrogen Bonds in Mixed Halide Perovskites
Source: J Phys Chem Lett. 2026 Apr 9;17(16):4755–63. doi: 10.1021/acs.jpclett.6c00523 (PMC13112441; doi:10.1021/acs.jpclett.6c00523)
Supplement: Supplementary file 1 [file jz6c00523_si_001.pdf]

SUPPORTING INFORMATION

Thermodynamic Stability and Hydrogen Bonds in Mixed Halide Perovskites

Liz Camayo-Gutierrez,[1] Javiera Ubeda,[1] Ana L. Montero-Alejo,[1,*]
Ricardo Grau-Crespo,[2] and Eduardo Menéndez-Proupin[3,†]

[1] *Departamento de Física, Facultad de Ciencias Naturales, Matemática y del Medio Ambiente (FCNMM), Universidad Tecnológica Metropolitana, José Pedro Alessandri 1242, Ñuñoa 7800002, Santiago, Chile*

[2] *School of Engineering and Materials Science, Queen Mary University of London, Mile End Road, London E1 4NS, UK*

[3] *Departamento de Física Aplicada I, Escuela Politécnica Superior, Universidad de Sevilla, Seville E-41011, Spain*

* amonteroa@utem.cl

† emenendez@us.es

I. ROTATIONAL ENTROPY OF MIXING FOR MIXED-CATION PEROVSKITES

This section derives a practical expression for the rotational entropy of mixing, $\Delta S_{\mathrm{mix}}^{\mathrm{rot}}$, in terms of species-specific rotational correlation times obtained from orientational autocorrelation functions.

The key idea is that any rotation-hindering potential barrier reduces the accessible phase space, lowering the entropy. One can estimate the entropy reduction from the effective energy barrier, which in turn can be estimated from the decay of the orientational auto-correlation function of the rotating cations, using relatively short molecular dynamics simulations.

To obtain an analytical expression for the orientational entropy loss, we map the reorientation of a given molecular axis into a one-dimensional (1D) rotor model in a periodic cosine potential,

$$V(\theta) = \frac{\Delta E}{2}\big(1 - \cos\theta\big), \tag{1}$$

where ΔE is the barrier height. The relevant dimensionless parameter is $y = \Delta E/(k_B T)$. The orientational partition function and entropy difference relative to a free rotor can be evaluated exactly[1]. The calculation involves modified Bessel functions I_0 and I_1:

$$\frac{\Delta S(y)}{k_B} = \ln I_0\Big(\frac{y}{2}\Big) - \frac{y}{2}\,\frac{I_1(y/2)}{I_0(y/2)}, \tag{2}$$

where $\Delta S(y) \equiv S_{\mathrm{orient}}(y) - S_{\mathrm{orient}}^{\mathrm{free}}$ is negative. We define the positive entropy-loss function

$$f(y) = -\Delta S(y) = k_B\left[-\ln I_0\Big(\frac{y}{2}\Big) + \frac{y}{2}\frac{I_1(y/2)}{I_0(y/2)}\right]. \tag{3}$$

The function $f(y)$ increases smoothly starting from zero (free rotation, $y \to 0$). The initial increase is very small, then becomes roughly linear, and finally saturates for very large barriers. When the barriers are non-negligible but not too high either (up to a few times $k_B T$, near the shaded area in the figure), $f(y)/k_B$ is well approximated as proportional to y, but with a slope much smaller than unity.

$$f(y)/k_B \approx \gamma\, y, \qquad \gamma \approx 0.185, \tag{4}$$

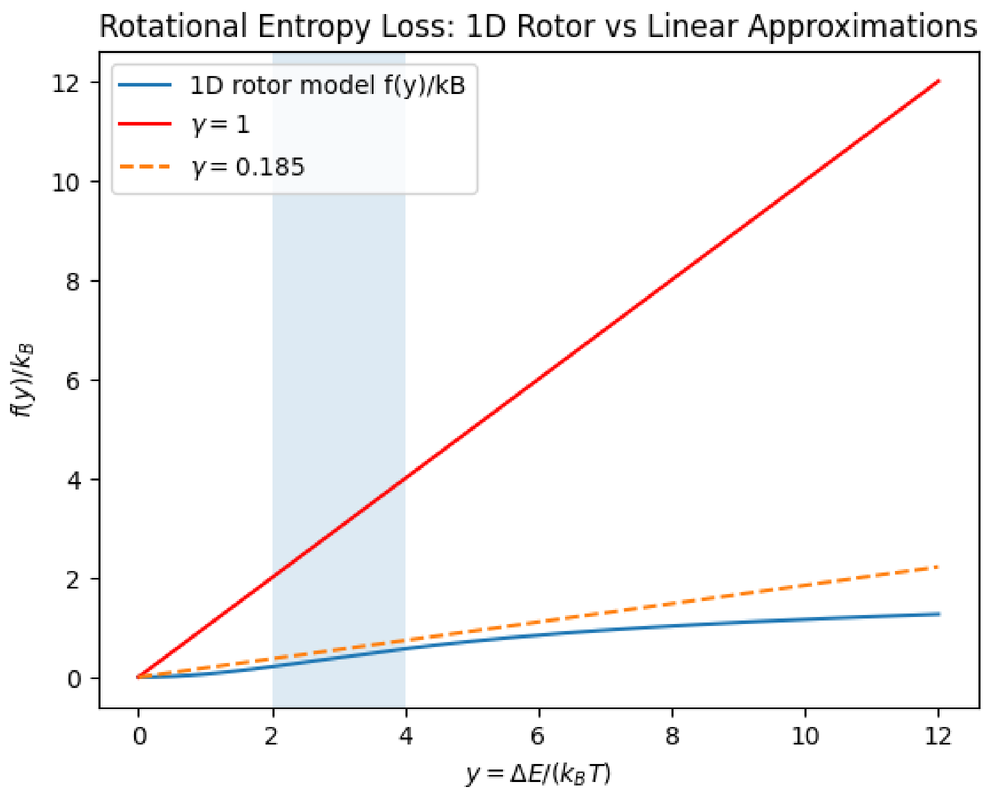

Figure S1. Normalized entropy loss $f(y)/k_B$ for the 1D periodic hindered rotor. The blue curve is the exact model [Eq. (4)], the red line is the naive linear approximation y, and the dashed black line is the best linear fit in the moderate barrier regime ($2 \le y \le 4$) with slope $\gamma \approx 0.185$. The shaded region highlights the range of y relevant to organic cations in hybrid perovskites.

where γ plays the role of an effective linear coefficient (much less than the naive value of unity). Figure S1 shows the exact $f(y)/k_B$ alongside the naive line (slope 1) and the effective linear line with slope $\gamma \approx 0.185$.

In an Arrhenius-like description of activated reorientation, the orientational correlation time τ of a molecular axis is related to the barrier height by

$$\tau \approx \tau_0\, e^{\Delta E/(k_B T)} = \tau_0\, e^{y}, \tag{5}$$

where τ_0 is a prefactor determined by librational dynamics. Taking the logarithm gives $y = \ln(\tau/\tau_0)$, therefore the entropy loss of a single cation can be expressed as:

$$\Delta S \approx -\gamma k_{\mathrm{B}} \ln(\tau/\tau_0) \tag{6}$$

We do not know τ_0, but for two environments (e.g. a cation in the mixture and in a pure end member), we take the difference, so the linear approximation leads to a cancellation of τ_0 and a simple expression for the rotational entropy change per cation:

$$\Delta S_{\mathrm{rot}}^{(\text{mix-pure})} \equiv -(f(y_{\mathrm{mix}}) - f(y_{\mathrm{pure}})) \approx -\gamma\, k_B\, \ln\left(\frac{\tau_{\mathrm{mix}}}{\tau_{\mathrm{pure}}}\right). \tag{7}$$

If, as happens in hybrid organic-inorganic perovskite solid solutions, mixing leads to additional hindering of rotation, the cation orientations will be more correlated over time and $\tau_{\mathrm{mix}} > \tau_{\mathrm{pure}}$, and therefore the rotational entropy of that molecular ion decreases upon mixing. Equation (7) is the final working formula used in our analysis. It relates the change in rotational entropy to correlation times.

The choice of a linear model with $\gamma \approx 0.185$ is, in summary, justified by the following arguments:

1. The reorientational dynamics of the molecular axis are governed by an effective potential along one reaction coordinate (the slowest mode), even though the full orientational motion is two-dimensional (in θ and ϕ). The effective coordinate can be modelled by the 1D periodic potential.

2. As seen above, the intermediate barrier regime relevant to room-temperature halide perovskites, the exact entropy loss function of this model is close to linear in y with a slope of $\gamma \approx 0.185$.

3. Using ratios of correlation times eliminates the unknown prefactor τ_0 in this approximation. The linear coefficient γ thus becomes an effective proportionality constant mapping kinetic barriers (from τ) to orientational entropy.

In conclusion, a simple hindered-rotor model yields a closed-form expression for the rotational contribution to the entropy of mixing in a solid solution with rotating ions, that can be readily evaluated with parameters extracted from molecular dynamics simulations.

In the moderate-barrier regime, the entropy loss is nearly linear in $y = \Delta E/(k_B T)$ with an effective slope $\gamma \approx 0.185$. Using the Arrhenius relation between barrier and correlation time, we derived Eq. (7), which expresses the change in rotational entropy upon mixing in terms of the ratio of relaxation times. This approach naturally removes the unknown attempt time τ_0 and permits a simple estimation of the rotational contribution to the mixing entropy.

II. COMPUTATIONAL METHODOLOGY

All *ab initio* molecular dynamics (AIMD) simulations were performed with the CP2K[2] package under NVT conditions at $T = 350$ K. Ionic forces were computed within density functional theory (DFT) using the Gaussian and plane-wave (GPW) formalism as implemented in the QUICKSTEP[3] module of CP2K. The Perdew–Burke–Ernzerhof (PBE)[4] exchange–correlation functional was employed, together with the DFT-D3[5] correction of Grimme to account for dispersion interactions. The Kohn–Sham valence orbitals were expanded in DZVP-MOLOPT[6] Gaussian basis sets for Pb, I, Br, C, N, and H, while core electrons were represented by Goedecker–Teter–Hutter (GTH[7,8]) pseudopotentials. The electronic minimization at each MD step was carried out using the orbital-transformation method[9,10].

The MD timestep was set to 1 fs. Temperature control was achieved using a Nos'e–Hoover chain thermostat of length 3. During the initial 2000 MD steps, a massive thermostat was applied, with one individual thermostat per atom and a time constant of 10 fs, in order to accelerate thermalisation in systems containing atoms with very different masses. After this initial stage, equilibration and production dynamics were performed using a time constant of 100 fs. For each mixed system, the corresponding pure end members were also simulated as reference states for the evaluation of mixing thermodynamic quantities. After thermalisation, each trajectory was propagated for 18 ps of production dynamics, and these production segments were used for the thermodynamic and hydrogen-bond analyses.

The mixing enthalpy at 350 K was approximated from time-averaged AIMD potential energies. Because all compared systems were simulated at the same temperature and the number of species is the same in the initial and final states, the kinetic-energy contribution to the mixing enthalpy is expected to largely cancel, and the pV term is negligible for the

condensed phase. The AIMD energy series was analyzed to obtain the time-averaged internal energy from the equilibrated part of the trajectory. The procedure computes the normalized autocorrelation function of the energy series using an FFT-based approach, and from this obtains the autocorrelation time. The procedure identifies a suitable starting point for the production region by scanning possible initial segments and selecting the one that maximizes the effective number of uncorrelated samples. Over this production region, it evaluates the mean energy, standard deviation, integrated autocorrelation time, effective sample size, and the standard error of the mean (SEM). These SEM values were subsequently propagated to estimate the uncertainty in the mixing enthalpy.

III. ADDITIONAL REORIENTATION DYNAMICS

In the main text we report reorientation dynamics using the representative molecular axis for each A-site cation (FA: N–N; MA: C–N) to define species-resolved rotational correlation times. For completeness, Fig. S2 summarizes the corresponding vector autocorrelation functions $C(t)$ for all studied compositions and for additional internal molecular vectors. These curves illustrate the anisotropy of molecular motion within the inorganic cage and confirm that the qualitative trends in reorientational dynamics across compositions are robust with respect to the specific choice of molecular axis.

Because $C(t)$ is normalized to $C(0) = 1$, we define the orientational correlation half-time $t_{1/2}$ as the time at which $C(t)$ first reaches 0.5, i.e., $C(t_{1/2}) = 0.5$, and use $t_{1/2}$ as a fit-free comparative measure of reorientation dynamics across systems. Using $t_{1/2}$ avoids imposing a specific functional form on $C(t)$, which can be ambiguous when multiple reorientation modes contribute or when the decay is non-exponential. All systems considered here reach $C(t) = 0.5$ within the simulated time window, enabling a consistent comparison of reorientation dynamics across compositions (see Table S1). In the rotational-entropy estimator, $t_{1/2}$ is used as a proxy for the characteristic reorientation time τ because only time ratios enter the analysis.

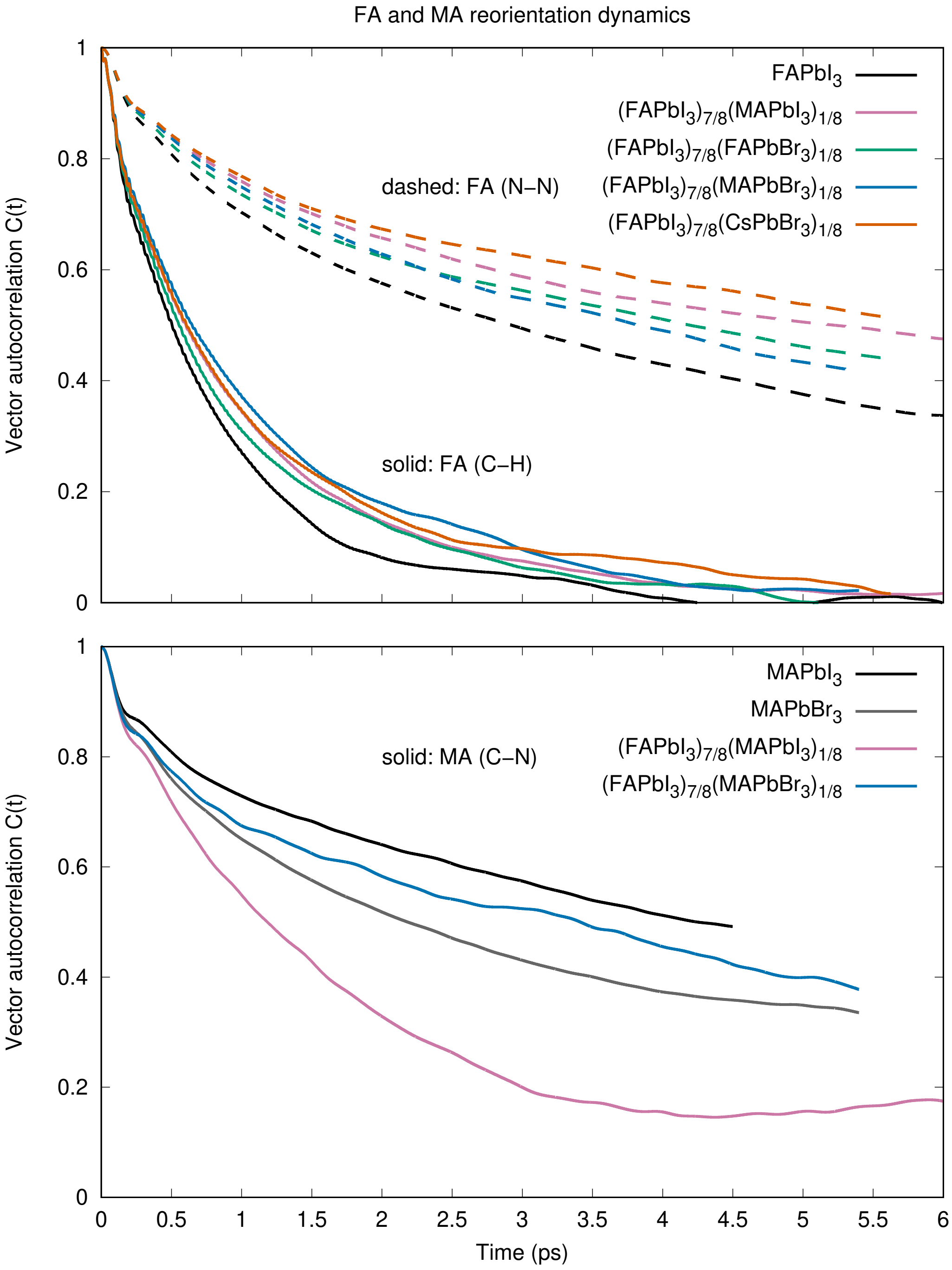

Figure S2. Vector autocorrelation functions $C(t)$ for A-site cation reorientation dynamics in the pure and mixed perovskites at 350 K. Top: FA-containing systems, comparing the FA N–N axis (dashed) and an additional FA internal C–H vector (solid). Bottom: MA-containing systems using the MA C–N axis. Autocorrelation functions were computed from AIMD trajectories and analyzed with TRAVIS[11].

Table S1. Orientational correlation half-time $t_{1/2}$ (ps) obtained from TRAVIS vector autocorrelation functions $C(t)$. For FA, $t_{1/2}$ corresponds to the N–N axis; for MA, $t_{1/2}$ corresponds to the C–N axis. Mixtures are reported for the composition $x = 1/8$ used in the AIMD calculations.

**Mixing site**	**System**	**FA (N–N)** $t_{1/2}$ (ps)	**MA (C–N)** $t_{1/2}$ (ps)
	$FAPbI_3$	2.9250	
Y site	$FAPbBr_3$	4.8340	
	$(FAPbI_3)_{7/8}(FAPbBr_3)_{1/8}$	4.1950	
	$FAPbI_3$	2.9250	
A site	$MAPbI_3$		4.2450
	$(FAPbI_3)_{7/8}(MAPbI_3)_{1/8}$	5.2260	1.1830
A and X site	$FAPbI_3$	2.9250	
	$CsPbBr_3$		
	$(FAPbI_3)_{7/8}(CsPbBr_3)_{1/8}$	6.0970	
	$FAPbI_3$	2.9250	
A and X site	$MAPbBr_3$		2.1820
	$(FAPbI_3)_{7/8}(MAPbBr_3)_{1/8}$	3.8280	3.4130

IV. ESTIMATION OF THERMODYNAMIC FUNCTIONS ERROR

The mixing enthalpy per ABY_3 formula unit was computed from the internal energies of the mixed and pure systems as

$$\Delta H_{\mathrm{mix}} = \frac{\langle U_{\mathrm{mix}}\rangle - x\langle U_A\rangle - (1-x)\langle U_B\rangle}{N_{\mathrm{fu}}}, \tag{8}$$

where U_{mix} is the total internal energy of the mixed supercell, U_A and U_B are the internal energies of the pure end members (e.g., $FAPbI_3$ and $FAPbBr_3$), x is the mole fraction of the substituent/minority endmember B in the mixture, and $N_{\mathrm{fu}} = 64$ is the number of ABY_3 formula units in the simulation cell. The internal energies U_{mix}, U_A, and U_B were extracted as time averages over the *ab initio* molecular dynamics trajectories. At fixed cell volume, we approximate $\Delta H_{\mathrm{mix}} \approx \Delta U_{\mathrm{mix}}$ per formula unit, as the pV contribution is negligible on this

scale.

Uncertainties were estimated from the energy time series by accounting for equilibration and time correlation, using an effective number of independent samples N_{eff} to compute the standard error of the mean, $\text{SEM}(U) = \sigma/\sqrt{N_{\text{eff}}}$, where σ is the standard deviation of the (production) energy time series. The uncertainty in ΔH_{mix} was obtained by standard error propagation from the SEMs of the mixed and reference energies and is reported as one standard deviation.

Assuming uncorrelated fluctuations of the three energy estimators, the uncertainty in the mixing energy

$$\Delta U_{\text{mix}} = U_{\text{mix}} - xU_A - (1-x)U_B \tag{9}$$

is obtained by standard linear error propagation as

$$\text{SEM}^2(\Delta U_{\text{mix}}) = \text{SEM}^2_{\text{mix}} + x^2\text{SEM}^2_A + (1-x)^2\text{SEM}^2_B, \tag{10}$$

where $\text{SEM}(\Delta U_{\text{mix}})$ is the standard error of the mean of ΔU_{mix}.

The corresponding uncertainty in the mixing enthalpy per formula unit follows directly from Eq. (8):

$$\text{SEM}(\Delta H_{\text{mix}}) = \frac{\text{SEM}(\Delta U_{\text{mix}})}{N_{\text{fu}}}. \tag{11}$$

The total Gibbs free energy of the mixed perovskites per ABY_3 formula unit is obtained as:

$$\Delta G^{\text{tot}}_{\text{mix}} = \Delta H_{\text{mix}} - T(\Delta S^{\text{conf}}_{\text{mix}} + \Delta S^{\text{rot}}_{\text{mix}}), \tag{12}$$

where ΔH_{mix} is the mixing enthalpy per formula unit, T is the simulation temperature, and $\Delta S^{\text{conf}}_{\text{mix}}$ and $\Delta S^{\text{rot}}_{\text{mix}}$ are the conformational and rotational entropies of mixing per formula unit.

Since $\Delta S^{\text{conf}}_{\text{mix}}$ is evaluated analytically, it carries no statistical uncertainty.

The rotational entropy of mixing is estimated from orientational correlation functions via the fit-free half-time $t_{1/2}$ (Table S1), which we use as an operational proxy for the characteristic reorientation time scale. Because $t_{1/2}$ is obtained from the first crossing $C(t_{1/2}) = 0.5$, it avoids imposing a specific functional form on $C(t)$. However, a rigorous statistical uncertainty for $\Delta S^{\text{rot}}_{\text{mix}}$ is still nontrivial to assign for the present AIMD trajectory lengths. In particular,

$t_{1/2}$ depends on the discrete sampling of $C(t)$, and exhibits block-to-block fluctuations for $\sim$10–20 ps trajectories.

Importantly, the rotational-entropy term is numerically small compared with the dominant configurational-entropy contribution and does not affect the qualitative thermodynamic conclusions. Consequently, even conservative variations in $\Delta S_{\mathrm{mix}}^{\mathrm{rot}}$ would not change the sign of $\Delta G_{\mathrm{mix}}^{\mathrm{tot}}$ nor the absence of a miscibility gap inferred from the curvature analysis. We therefore report $\Delta S_{\mathrm{mix}}^{\mathrm{rot}}$ as a best estimate and do not propagate a formal uncertainty for this term.

Since $\Delta S_{\mathrm{mix}}^{\mathrm{conf}}$ is analytical and we do not propagate uncertainties for $\Delta S_{\mathrm{mix}}^{\mathrm{rot}}$, the reported uncertainty of the total mixing free energy reduces to

$$\mathrm{SEM}(\Delta G_{\mathrm{mix}}^{\mathrm{tot}}) \approx \mathrm{SEM}(\Delta H_{\mathrm{mix}}). \tag{13}$$

V. REGULAR SOLUTION ANALYSIS

To assess the stability of the solid solutions against phase separation, we analyzed the curvature of the mixing free energy within a regular-solution model, parameterized using the AIMD-evaluated thermodynamic quantities at $x = 1/8$ (Fig. S3).

In the regular-solution approximation, the enthalpy of mixing per $\mathrm{ABY_3}$ formula unit is represented by a composition-independent bowing parameter W,

$$\Delta H_{\mathrm{mix}}(x) = W\, x(1-x), \tag{14}$$

with

$$W = \frac{\Delta H_{\mathrm{mix}}(x)}{x(1-x)}. \tag{15}$$

The configurational entropy of mixing is treated in the ideal-solution approximation on the relevant mixed sublattice(s),

$$\Delta S_{\mathrm{mix}}^{\mathrm{conf}}(x) = -n_s k_B \left[x \ln x + (1-x)\ln(1-x)\right], \tag{16}$$

where n_s is the number of mixed sites per $\mathrm{ABY_3}$ formula unit ($n_s = 1$ for A-mixing, $n_s = 3$

for Y-mixing, and $n_s = 4$ for simultaneous A+Y mixing).

For systems containing molecular A-site cations, we include an additional rotational-entropy correction using the same symmetric regular-solution form as in the main text,

$$-T\Delta S_{\text{mix}}^{\text{rot}}(x) = T\lambda\, x(1-x), \tag{17}$$

where λ is determined from the AIMD-evaluated rotational entropy of mixing at x:

$$\lambda = \frac{-\Delta S_{\text{mix}}^{\text{rot}}(x)}{x(1-x)}. \tag{18}$$

With this definition, $\lambda > 0$ corresponds to a loss of rotational entropy upon mixing (i.e., $\Delta S_{\text{mix}}^{\text{rot}} < 0$).

Combining the enthalpic and rotational contributions leads to an effective regular-solution parameter,

$$W_{\text{eff}} = W + T\lambda, \tag{19}$$

and the corresponding total mixing free energy becomes

$$\Delta G_{\text{mix}}^{\text{tot}}(x) = W_{\text{eff}}x(1-x) + n_s k_B T \left[x \ln x + (1-x)\ln(1-x)\right]. \tag{20}$$

For comparison, the configurational-only free energy is recovered by setting $\lambda = 0$ (i.e., $W_{\text{eff}} = W$), yielding $\Delta G_{\text{mix}}^{\text{conf}}(x)$.

Within this model, a miscibility gap occurs when the curvature of $\Delta G_{\text{mix}}(x)$ becomes negative, which leads to the condition

$$W_{\text{eff}} > 2n_s k_B T. \tag{21}$$

The resulting composition-dependent profiles of ΔH_{mix}, $-T\Delta S_{\text{mix}}^{\text{conf}}$, $-T(\Delta S_{\text{mix}}^{\text{conf}}+\Delta S_{\text{mix}}^{\text{rot}})$, and the corresponding $\Delta G_{\text{mix}}^{\text{conf}}$ and $\Delta G_{\text{mix}}^{\text{tot}}$ are shown in Fig. S3.

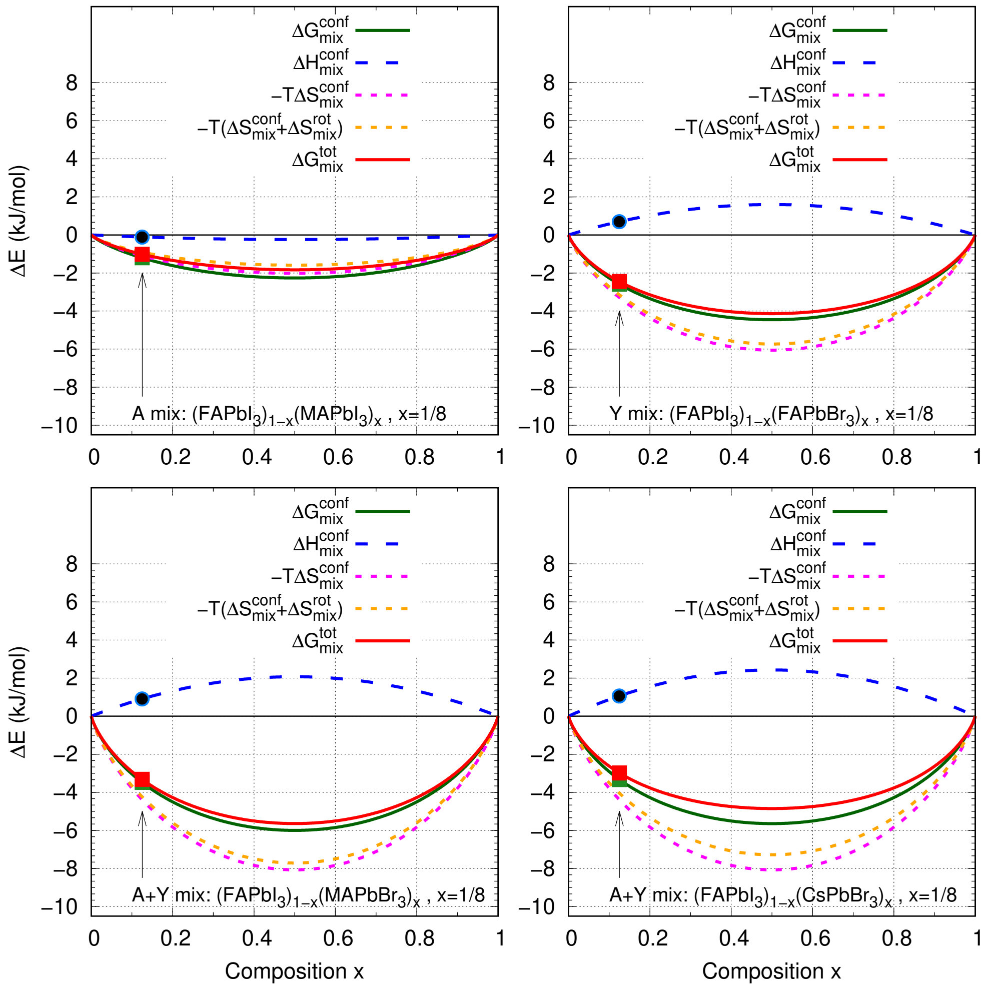

Figure S3. Regular-solution model analysis of the mixing free energy at 350 K. Shown are $\Delta H_{\text{mix}}(x) = Wx(1-x)$, the configurational entropy term $-T\Delta S_{\text{mix}}^{\text{conf}}(x)$ with n_s mixed sites per formula unit, the combined entropic term $-T(\Delta S_{\text{mix}}^{\text{conf}} + \Delta S_{\text{mix}}^{\text{rot}})$ using $-T\Delta S_{\text{mix}}^{\text{rot}}(x) = T\lambda x(1-x)$, and the corresponding free energies $\Delta G_{\text{mix}}^{\text{conf}}(x)$ and $\Delta G_{\text{mix}}^{\text{tot}}(x)$. Parameters W and λ are obtained from the AIMD-evaluated thermodynamic quantities at $x = 1/8$.

VI. HYDROGEN BOND ANALYSIS

Figures S4–S5 report the CDF maps of $d(\text{H}-Y)$ versus $\measuredangle(\text{N}-\text{H}-Y)$ (with $Y = \text{I}, \text{Br}$) for the mixed and pure perovskites. In all cases, the geometrical hydrogen-bond (HB) definition used in the main text ($d(\text{H}-Y) \leq 3$ Å and $135^\circ \leq \measuredangle(\text{N}-\text{H}-Y) \leq 180^\circ$) encloses the high-probability region of the CDFs, supporting its transferability across compositions for

extracting HB dynamical properties.

To complement the main-text discussion of HB lifetimes, Figures S6–S8 report the N−H$\cdots$I and N−H$\cdots$Br HB autocorrelation functions (ACFs) computed from the AIMD trajectories for representative mixed and pure systems. For each HB type we show both the continuous ACF (solid line), which counts only uninterrupted HBs, and the intermittent ACF (dotted line), which allows HB breaking and reforming. The ACF decays were fitted using sums of exponentials (1–5 terms) to extract characteristic HB lifetimes and to assess the robustness of the fitted time scales with respect to the fitting form. For convenience, Fig. S9 compiles the resulting ACFs for all systems together.

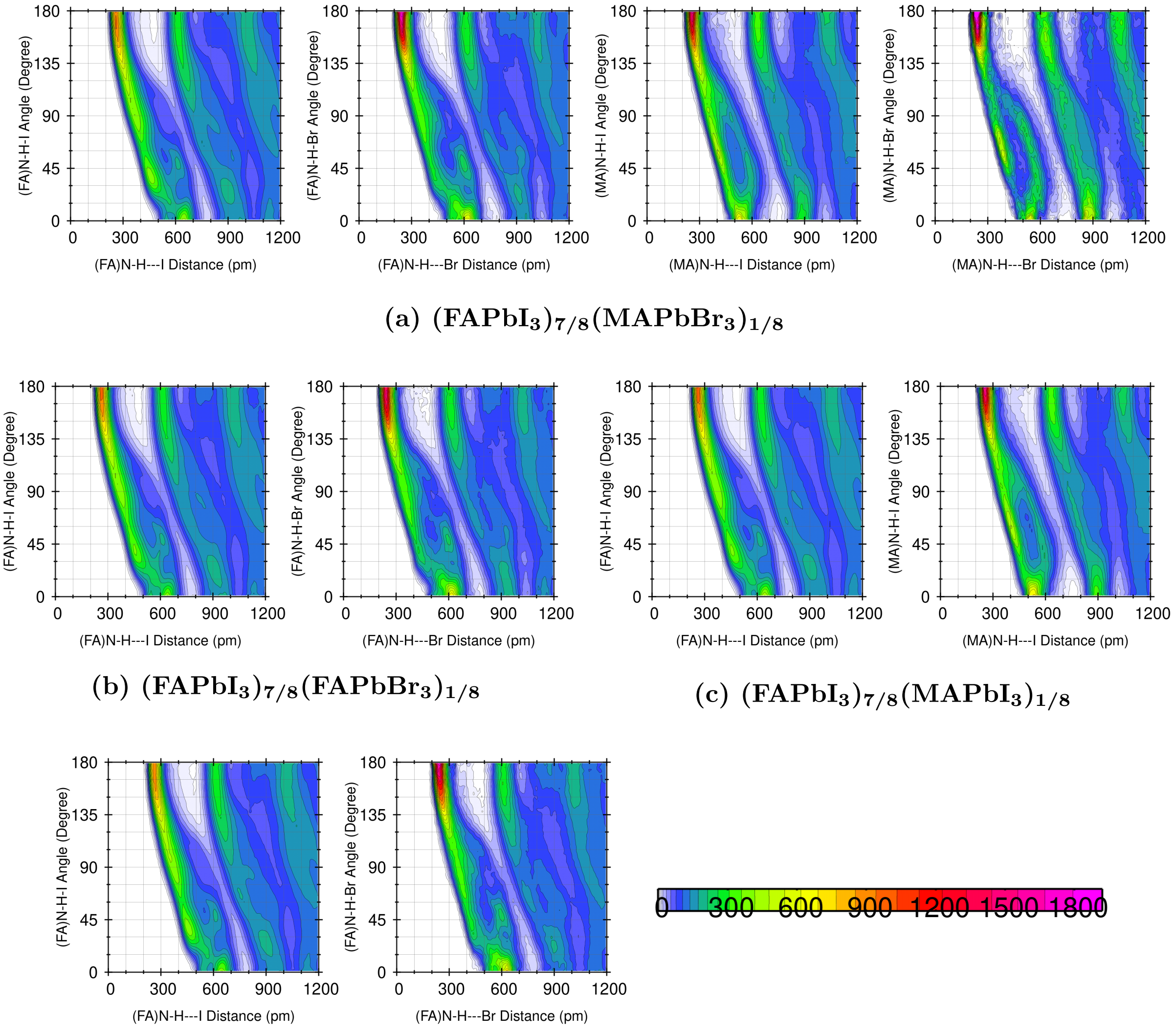

(a) $(FAPbI_3)_{7/8}(MAPbBr_3)_{1/8}$

(b) $(FAPbI_3)_{7/8}(FAPbBr_3)_{1/8}$

(c) $(FAPbI_3)_{7/8}(MAPbI_3)_{1/8}$

(d) $(FAPbI_3)_{7/8}(CsPbBr_3)_{1/8}$

Figure S4. Combined distribution functions (CDFs) for N−H···I and N−H···Br hydrogen bonds (HBs) in the studied perovskites. The CDFs are shown as 2D histograms where the color scale indicates the configuration frequency in each of the 300 × 300 bins (proportional to the configuration probability). Panels (a–d) correspond to the mixed compounds: (a) $(FAPbI_3)_{7/8}(MAPbBr_3)_{1/8}$, (b) $(FAPbI_3)_{7/8}(FAPbBr_3)_{1/8}$, (c) $(FAPbI_3)_{7/8}(MAPbI_3)_{1/8}$, and (d) $(FAPbI_3)_{7/8}(CsPbBr_3)_{1/8}$. In each mixed system, the N−H···I and N−H···Br CDFs are reported separately for FA and MA whenever present; for $(FAPbI_3)_{7/8}(CsPbBr_3)_{1/8}$ only FA contributes.

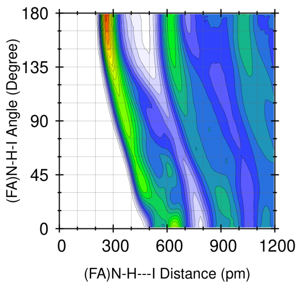

(a) FAPbI$_3$

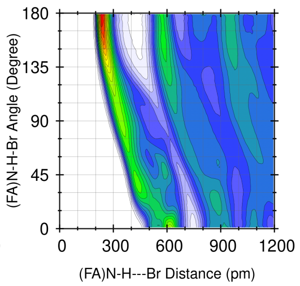

(b) FAPbBr$_3$

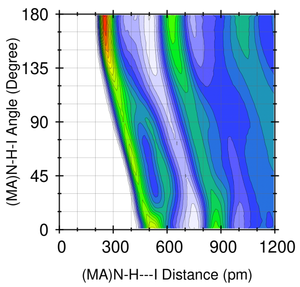

(c) MAPbI$_3$

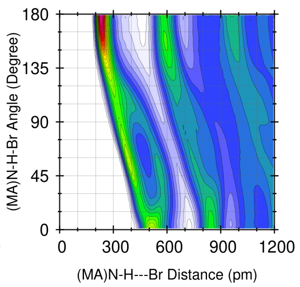

(d) MAPbBr$_3$

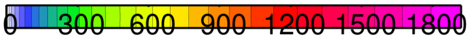

(e) z axis box

Figure S5. Combined distribution functions (CDFs) for N−H$\cdots$I and N−H$\cdots$Br hydrogen bonds (HBs) in the studied perovskites. The CDFs are shown as 2D histograms where the color scale indicates the configuration frequency in each of the 300 × 300 bins (proportional to the configuration probability). Panels (a–d) show the pure end members corresponding to the solid solutions of the previous figure.

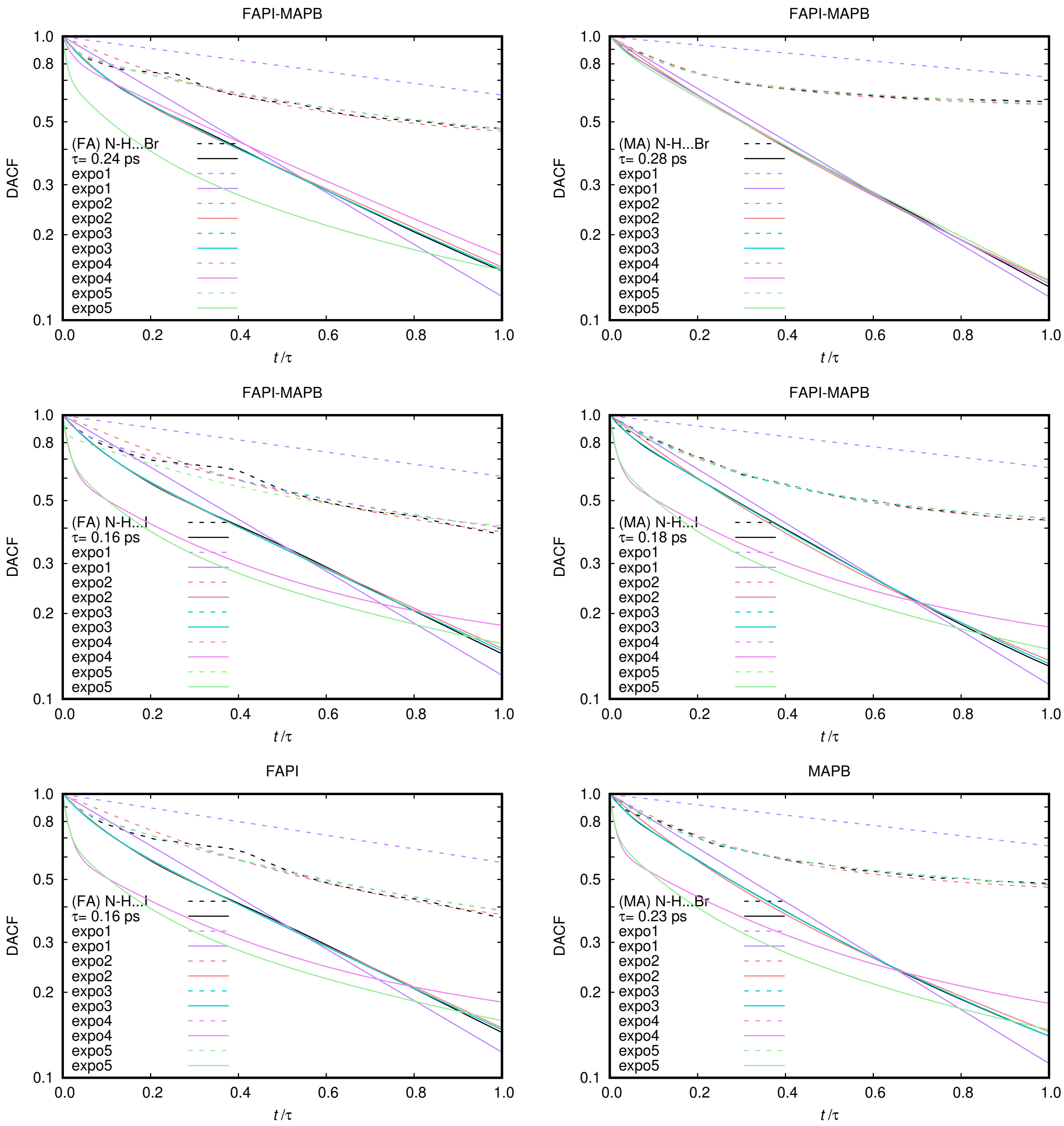

Figure S6. Hydrogen-bond autocorrelation functions (ACFs) for N−H$\cdots$I and N−H$\cdots$Br HBs in $(FAPbI_3)_{7/8}(MAPbBr_3)_{1/8}$, $FAPbI_3$, and $MAPbBr_3$. Solid lines (black): continuous ACF; dotted lines (black): intermittent ACF. Colored curves show poly-exponential fits using 1–5 exponential terms (as indicated in the legend) applied to the continuous and intermittent ACF decay. These ACFs underpin the HB lifetime analysis discussed in the main text.

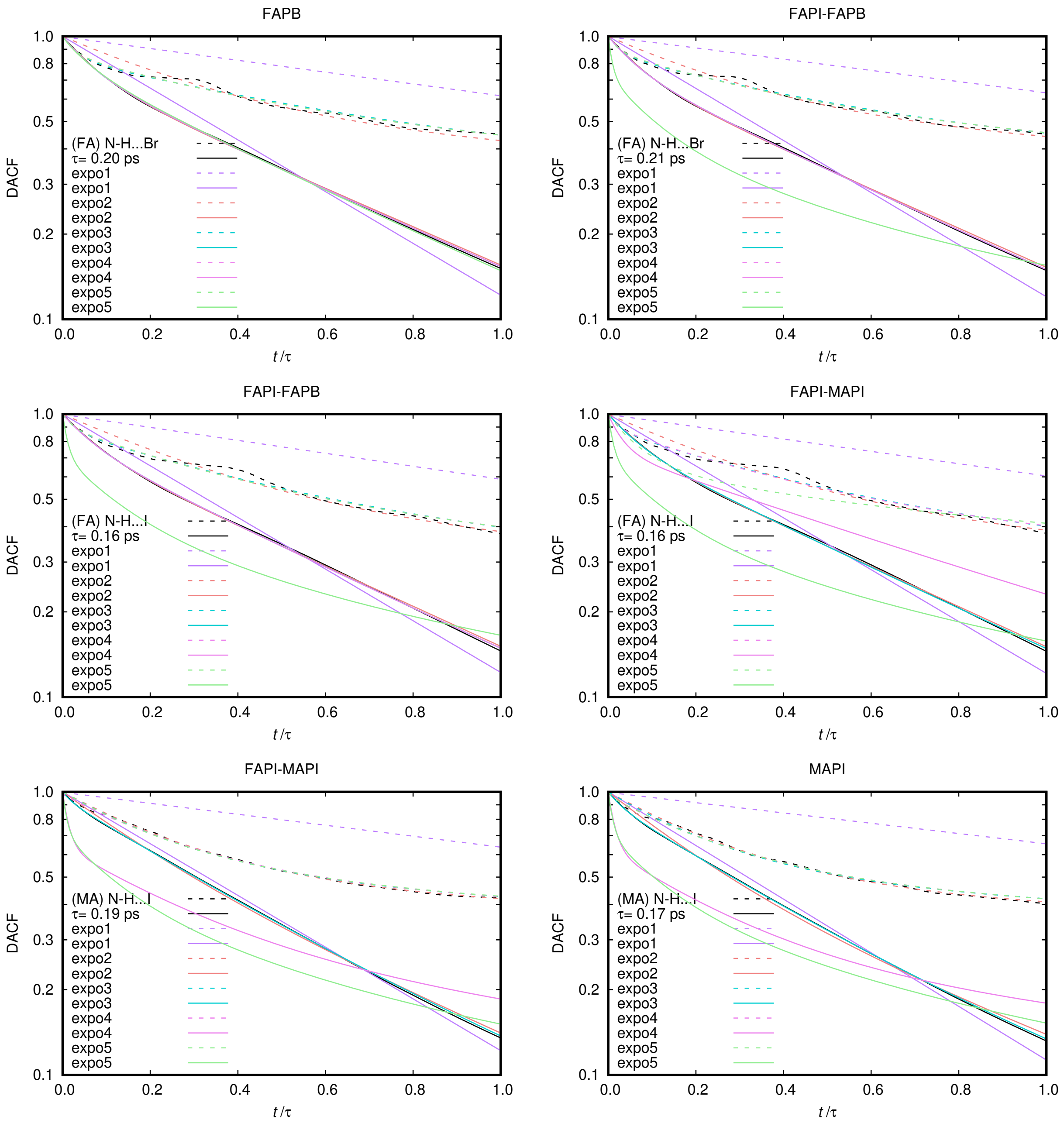

Figure S7. Hydrogen-bond autocorrelation functions (ACFs) for N−H···I and/or N−H···Br HBs in $FAPbBr_3$, $(FAPbI_3)_{7/8}(FAPbBr_3)_{1/8}$, $(FAPbI_3)_{7/8}(MAPbI_3)_{1/8}$, $MAPbI_3$, $FAPbBr_3$. Solid lines (black): continuous ACF; dotted lines (black): intermittent ACF. Poly-exponential fits using 1–5 exponential terms (as indicated in the legend) applied to the continuous and intermittent ACF decay. These ACFs underpin the HB lifetime analysis discussed in the main text.

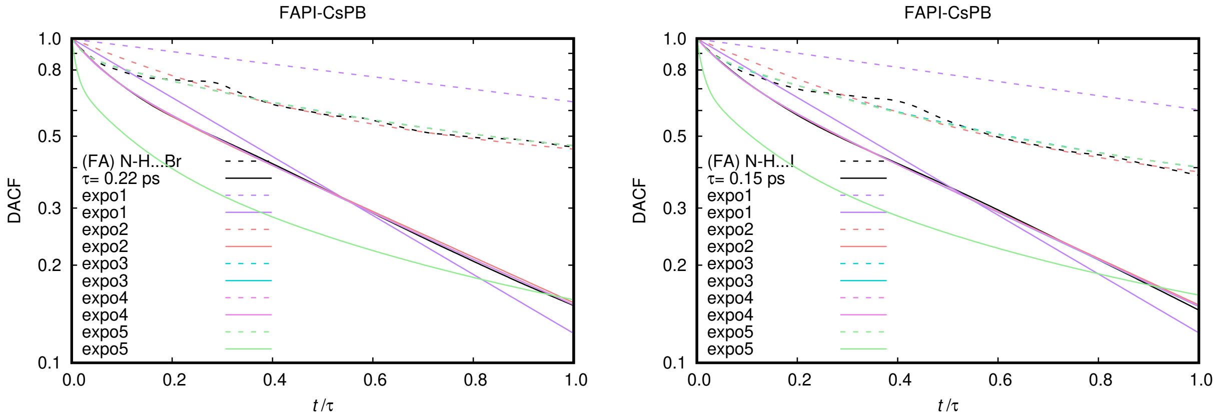

Figure S8. Hydrogen-bond autocorrelation functions (ACFs) for N−H$\cdots$I and N−H$\cdots$Br HBs in $(FAPbI_3)_{7/8}(CsPbBr_3)_{1/8}$. Solid line (black): continuous ACF; dotted line (black): intermittent ACF. Poly-exponential fits using 1–5 exponential terms (as indicated in the legend) applied to the continuous and intermittent ACF decay. These ACFs underpin the HB lifetime analysis discussed in the main text.

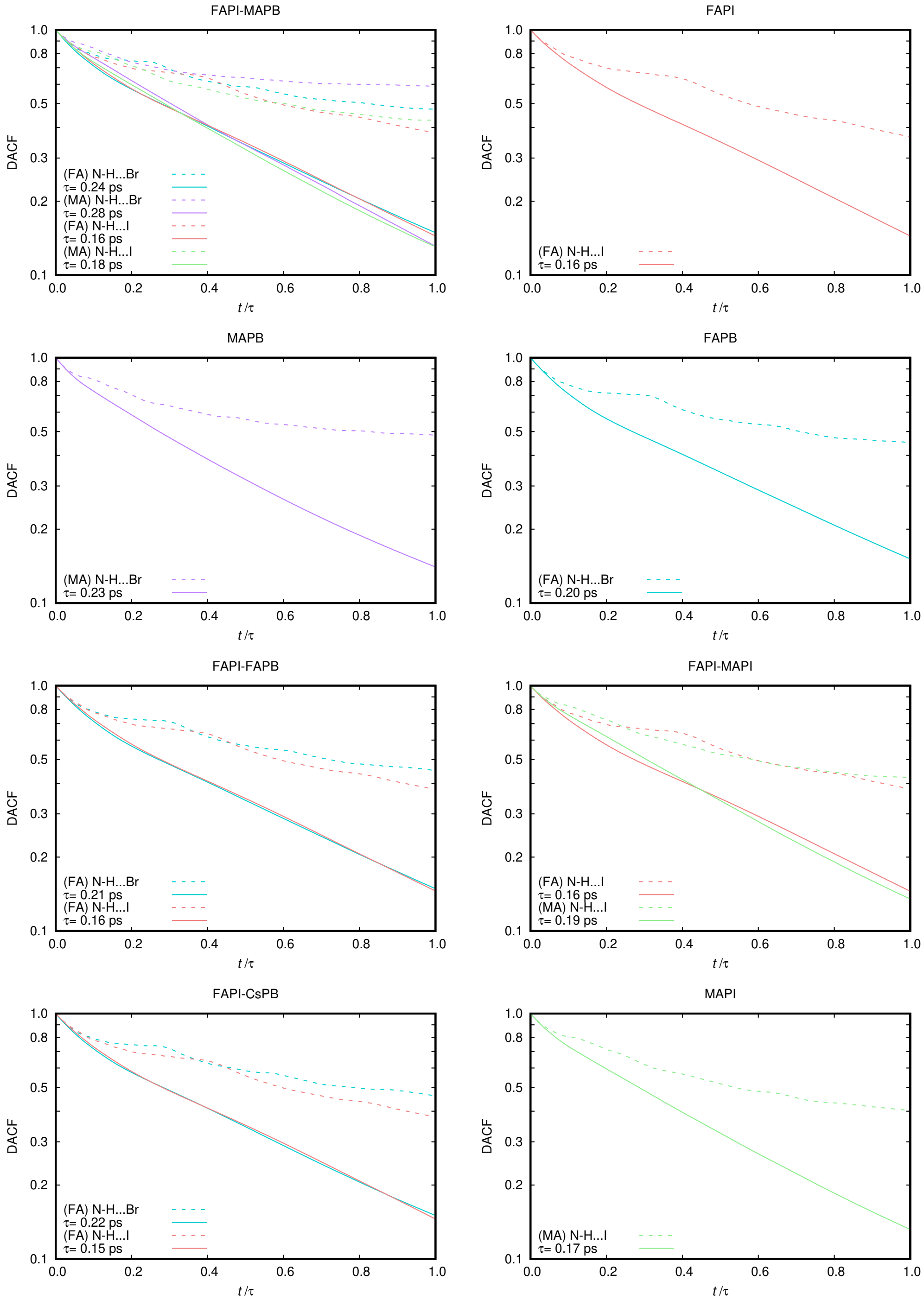

Figure S9. Hydrogen-bond autocorrelation functions (ACFs) for all studied systems. Aggregate N−H$\cdots$I and N−H$\cdots$Br ACFs are shown for $(FAPbI_3)_{7/8}(MAPbBr_3)_{1/8}$, $FAPbI_3$, $MAPbBr_3$, $FAPbBr_3$, $(FAPbI_3)_{7/8}(FAPbBr_3)_{1/8}$, $(FAPbI_3)_{7/8}(MAPbI_3)_{1/8}$, $(FAPbI_3)_{7/8}(CsPbBr_3)_{1/8}$, and $MAPbI_3$. Solid lines correspond to the continuous ACF (uninterrupted HBs), and dotted lines to the intermittent ACF (allowing HB breaking and reforming).

VII. ROOT MEAN SQUARE DISPLACEMENT (RMSD) ANALYSIS

Figure S10 shows RMSD-time curves reported for atomic species (Pb, I, Br) and by A-site components (FA, MA, Cs), for the pure and mixed perovskite compositions. For molecular cations, RMSD was computed for the center of mass (COM), whereas for inorganic species it was computed for the atomic positions. These comparisons were used to examine whether local mobility trends correlate with the thermodynamic signatures discussed in the main text.

Across all compositions, the dynamics of the Pb sublattice are essentially unchanged by mixing, indicating that mixing does not disrupt the inorganic cage on the timescale probed by AIMD. Iodide motion is also nearly invariant across the studied alloys. In MA-containing systems, the RMSD of the MA COM closely follows that of iodide, consistent with stronger and more persistent MA–I interactions. In contrast, the FA COM RMSD deviates more substantially from that of I, consistent with weaker and less persistent FA–I hydrogen bonds and greater orientational freedom. This dynamical asymmetry is consistent with the heterogeneous reorientational response observed in FA/MA mixtures and highlights that local mobility differences do not map trivially onto the mixing enthalpy, and may contribute to the rotational-entropy penalty discussed in the main text.

In the Br–FA panel, the FA COM RMSD is smaller in pure $FAPbBr_3$ than in the Br-containing mixed compositions, whereas the RMSD of Br remains nearly invariant across the same set of systems. This suggests that FA is more spatially confined in a homogeneous Br environment, while I/Br chemical disorder leads to a more heterogeneous local environment and thus to larger FA displacements. Importantly, this enhanced FA mobility in the mixed systems does not correlate directly with their thermodynamic stability, supporting the conclusion that hydrogen-bond dynamics are not the primary driver of alloy stabilization.

In $(FAPbI_3)_{7/8}(CsPbBr_3)_{1/8}$, the FA COM and Cs RMSD curves have comparable amplitudes, indicating that the two A-site species coexist dynamically within the same inorganic framework. This contrasts with FA–MA mixtures, where differences in size, geometry, and interaction strengths can lead to a more heterogeneous A-site dynamical response. Importantly, the comparable mobility of FA and Cs does not imply a reduced enthalpy of mixing; rather, $(FAPbI_3)_{7/8}(CsPbBr_3)_{1/8}$ remains thermodynamically favorable at 350 K because con-

figurational entropy dominates the free-energy balance despite a positive ΔH_{mix}, consistent with the thermodynamic analysis in the main text.

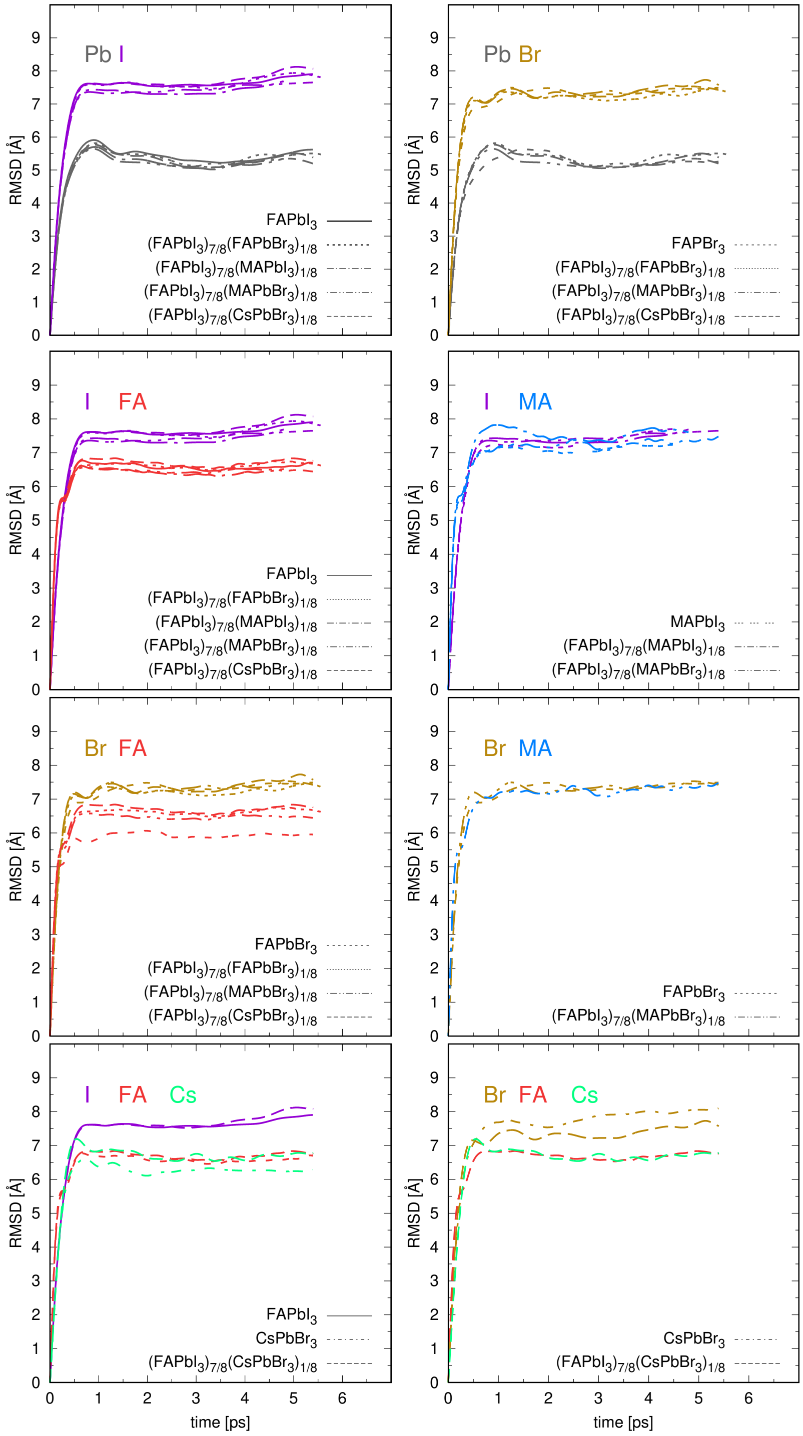

Figure S10. RMSD–time curves grouped by species (Pb, I, Br) and A-site components (FA, MA, Cs) for the pure and mixed perovskite compositions. For FA/MA, RMSD corresponds to the molecular center of mass (COM). All curves were computed over the same production trajectory window.

REFERENCES

(1) Pitzer, K. S.; Gwinn, W. D. Energy Levels and Thermodynamic Functions for Molecules with Internal Rotation I. Rigid Frame with Attached Tops. *J. Chem. Phys.* **1942**, 10 (7), 428 – 440.

(2) Kühne, T. D.; Iannuzzi, M.; Del Ben, M.; Rybkin, V. V.; Seewald, P.; Stein, F.; Laino, T.; Khaliullin, R. Z.; Schütt, O.; Schiffmann, F.; et al. CP2K: An Electronic Structure and Molecular Dynamics Software Package -Quickstep: Efficient and Accurate Electronic Structure Calculations. J. Chem. Phys. 2020, 152 (19).

(3) VandeVondele, J.; Krack, M.; Mohamed, F.; Parrinello, M.; Chassaing, T.; Hutter, J. Quickstep: Fast and Accurate Density Functional Calculations Using a Mixed Gaussian and Plane Waves Approach. Comput. Phys. Commun. 2005, 167 (2), 103–128.

(4) Perdew, J.; Burke, K.; Ernzerhof, M. Generalized Gradient Approximation Made Simple. Phys. Rev. Lett. 1996, 77 (18), 3865–3868.

(5) Grimme, S.; Antony, J.; Ehrlich, S.; Krieg, H. A Consistent and Accurate Ab Initio Parametrization of Density Functional Dispersion Correction (DFT-D) for the 94 Elements H-Pu. J. Chem. Phys. 2010, 132 (15), 154104.

(6) VandeVondele, J.; Hutter, J. Gaussian Basis Sets for Accurate Calculations on Molecular Systems in Gas and Condensed Phases. J. Chem. Phys. 2007, 127 (11), 114105.

(7) Goedecker, S.; Teter, M.; Hutter, J. Separable Dual-Space Gaussian Pseudopotentials. Phys. Rev. B 1996, 54 (3), 1703–1710.

(8) Krack, M. Pseudopotentials for H to Kr Optimized for Gradient-Corrected Exchange-Correlation Functionals. Theor. Chem. Acc. 2005, 114 (1–3), 145–152.

(9) VandeVondele, J.; Hutter, J. An Efficient Orbital Transformation Method for Electronic Structure Calculations. J. Chem. Phys. 2003, 118 (10), 4365–4369.

(10) Weber, V.; VandeVondele, J.; Hutter, J.; Niklasson, A. M. N. Direct Energy Functional Minimization under Orthogonality Constraints. J. Chem. Phys. 2008, 128 (8), 84113.

(11) Brehm, M.; Thomas, M.; Gehrke, S.; Kirchner, B. TRAVIS—A Free Analyzer for Trajectories from Molecular Simulation. *J. Chem. Phys.* **2020**, 152 (16), 164105.
